# Translating the EORTC CAT core and the QLQ-C30 to the EQ-5D-5L in patients with metastatic breast cancer: A comparison of direct and indirect mapping algorithms

**DOI:** 10.1007/s10198-025-01824-0

**Published:** 2025-08-21

**Authors:** Pimrapat Gebert, Anna Maria Hage, Felix Fischer, Christoph Paul Klapproth, Ulrike Grittner, Maria Margarete Karsten

**Affiliations:** 1 Berlin Institute of Health at Charité–Universitätsmedizin Berlin, Berlin, Germany; 2 Corporate member of Freie Universität Berlin and Humboldt-Universität zu Berlin, Institute of Biometry and Clinical Epidemiology, Charité– Universitätsmedizin Berlin, Berlin, Germany; 3 Corporate Member of Freie Universität Berlin and Humboldt Universität zu Berlin, Department of Gynecology with Breast Center, Charité– Universitätsmedizin Berlin, Berlin, Germany; 4 Corporate member of Freie Universität Berlin and Humboldt-Universität zu Berlin, Department of Psychosomatic Medicine, Center for Patient Centered Outcomes Research, Charité– Universitätsmedizin Berlin, Berlin, Germany; 5 BIH Charité Digital Clinician Scientist Program, Berlin Institute of Health at Charité– Universitätsmedizin Berlin, BIH Biomedical, Innovation Academy, Berlin, Germany

**Keywords:** Mapping, Patient-reported outcome, Advanced breast cancer, EQ-5D-5L, EORTC QLQ-C30, EORTC CAT core

## Abstract

**Background:**

To enable the use of different non-preference-based patient-reported outcome measures to derive utility values for health economic evaluations in oncological trials, this study developed direct and indirect mapping algorithms for estimating the EQ-5D-5L utility index via the German value set from the EORTC CAT Core and the QLQ-C30 in metastatic breast cancer patients.

**Methods:**

We included 1,839 observations from 878 patients with metastatic breast cancer from the PRO B study. We compared direct mapping algorithms, including adjusted limited dependent variable mixture models (ALDVMM), Tobit regression, ordinal least squares regression, and adjusted beta regression, while indirect mapping employed a generalized ordered logit model. Visualization was used to assess model performance across the entire distribution, while quantitative evaluation was performed using mean absolute error (MAE), root mean squared error (RMSE), and mean prediction bias.

**Results:**

Among the direct algorithms, adjusted beta regression demonstrated the best performance. It had the lowest MAE of 0.07–0.08 and RMSE of 0.11–0.13, a mean prediction bias of -0.004, close to zero. The indirect mapping model also performed well, with a mean prediction bias of 0.04 and MAE of 0.07, showing performance comparable to the preferred direct mapping algorithm for both the EORTC CAT Core and the QLQ-C30.

**Conclusions:**

This study developed and validated robust direct and indirect algorithms for estimating the EQ-5D-5L utility index from the EORTC CAT Core and the QLQ-C30 based on the German tariff. In particular, using this indirect mapping algorithm, the EORTC CAT Core and QLQ-C30 can be translated into quality-adjusted life-years, facilitating health economic evaluations across different country tariffs.

**Trial registration:**

DRKS (German Clinical Trials Register) DRKS00024015. Registered on 15 February 2021, https//drks.de/search/de/trial/DRKS00024015.

**Supplementary Information:**

The online version contains supplementary material available at 10.1007/s10198-025-01824-0.

## Introduction

Quality-adjusted life years (QALYs) are a common health outcome metric frequently used in cost-effectiveness analyses in clinical studies. Health utility data, which serve as weights for quality of life (QoL), are required for calculating QALYs. Unfortunately, instruments that provide utility information—such as the EuroQoL 5-Dimension (EQ-5D) with 5 levels (EQ-5D-5L)—are not always administered or prioritized in clinical trials. Instead, health-related quality of life (HRQoL) data from oncology trials are most commonly collected via disease-specific instruments, such as the European Organization for Research and Treatment of Cancer (EORTC) Quality of Life Questionnaire (QLQ-C30), a nonpreference-based measure of patient-reported outcomes (PROs).

Recently, the EORTC developed the EORTC Quality of Life Utility—Core 10 Dimensions (QLU-C10D) [1] a cancer-specific preference-based measure designed to generate utilities from the QLQ-C30, with various tariffs available. Despite the availability of cancer-specific utility measures such as the QLU-C10D, mapping HRQoL scores from nonpreference-based measures to preference-based ones—such as mapping the EQ-5D from the QLQ-C30—remains essential. This is because the EQ-5D is widely recognized and commonly used as a standard in economic evaluations, facilitating comparability across various diseases and interventions. Additionally, mapping enables the integration of cancer-specific HRQoL data into broader health economic models, making it easier to apply these data in diverse healthcare contexts.

Several studies have developed mapping algorithms to translate the EQ-5D from the QLQ-C30 in cancer patients (Table 1). These studies employed various methods, including ordinal least squares (OLS), adjusted limited dependent variable mixture models (ALDVMMs), censored least absolute deviation, Tobit regression, and beta regression for direct mapping. However, these studies reported bias, with values being underestimated for healthier patients and overestimated for those in poor health. Comparisons of different models showed that mixture models performed better for patients with poor health, while OLS was more accurate for healthier individuals. Adjusted beta regression has emerged as a strong method for handling skewed and multimodal data in cancer-related quality of life research.

**Table 1:** Summary literatures developed mapping algorithms to translate the EQ-5D from the QLQ-C30 in cancer patients

Study	Sample	EQ-5D	Number of observations	Mapping method	Key findings
Crott and Briggs [2]	Breast cancer, multicenter (Belgium, France, the Netherlands, Switzerland, and the UK)	3L	870	OLS regression	OLS regression using PF, EF, SF, PA, SL, CO, DI, and its square (Average error 0.0602, RMSE 0.096).
Kim et al. [3]	Metastatic breast cancer, Korea	3L	199	OLS regression	Using all the QLQ-C30 domains showed the best performance compared to the model that included demographics (age, sex, and ECOG score) (MAE 0.092).
Khan and Morris [4]	Non-small cell lung cancer patients	3L	2038	- Linear mixed model - Tobit mixed model - Quadratic mixed model - Quantile fixed effects model - Censored Least Absolute Deviation (CLAD) - Beta binomial regression mixed model	Beta binomial regression (MAE 0.10, RMSE 0.09) using all domains of the QLQ-C30 as predictors.
Khan et al. [5]	Non-small cell lung cancer patients	3L and 5L	98	- Linear random effects model - ALDVMM - Beta binomial model	Beta binomial model was the best performing model (MAE 0.075, RMSE 0.092).
Woodcock et al. [6]	20 different tumor types	3L	3866	*Direct mapping* - OLS regression - ALDVMM - One-part beta - Two-part beta *Indirect mapping* - Ordinal logistic regression - Multinomial logistic regression	Two-part beta was the best performing (MAE 0.117, RMSE 0.109) Ordinal logistic regression (MAE 0.116, RMSE 0.112) Multinomial logistic regression (MAE 0.115, RMSE 0.110) The multinomial response mapping algorithm performed well on the low EQ-5D-3 L scores, and the OLS and logistic algorithm performed well on the high EQ-5D-3 L scores.
Beck et al. [7]	Head and neck cancer	3L	361	- OLS regression - Mixed effects model - Cox regression with censoring of all EQ-5D utility index scores < 1. - Beta regression	The beta regression model showed best model fit, with QL, PF, RF, EF, PA domains as predictors (MAE 0.0949, RMSE 0.1209).
Ameri et al. [8]	Colorectal cancer	5L	252	- OLS regression - Tobit regression - Censored least absolute deviation (CLAD)	The OLS model using QL, PF, EF, PA, and SL domains showed the best performance (MAE 0.0932, RMSE 0.129).
Hagiwara et al. [9]	Local advanced, metastatic cancers (lung, stomach, colorectal, breast cancer)	5L	903	*Direct mapping* - OLS regression - Beta regression - Tweedie regression - Tobit regression - Two-part linear regression - Two-part beta regression *Indirect mapping* - Ordinal logistic regression	Two-part beta regression for direct mapping (MAE 0.075, RMSE 0.099) Ordinal logistic regression for indirect mapping (MAE 0.090, RMSE 0.100)
Yousefi et al. [10]	Colorectal and breast cancer	5L	668	- OLS regression - CLAD models	OLS regression with QL, PF, RF, EF, FA, PA, interaction (QL*FA, PF*RF, RF*PA), and age as predictors (MAE 0.0712, RMSE 0.1002)
Gray et al. [11]	HER2-positive advanced breast cancer	3L	3766	- OLS regression - ALDVMM - Ordinal probit models	ALDVMM with 4 components was the best performance for direct mapping (MAE 0.1173, RMSE 0.1675). Ordinal probit models for indirect mapping (MAE 0.119, RMSE 0.1702) using all domains and age as predictors.
Meunier et al. [12]	Mixed cancer, multicenter	5L	692	*Direct mapping* - OLS regression - Tobit regression - Two-part beta regression - Mixture beta regression - ALDVMM *Indirect mapping* - Ordinal logistic regression	Two-part beta regression for direct mapping (MAE 0.0701, RMSE 0.0950) Ordinal logistic regression for indirect mapping (MAE 0.0708, RMSE 0.0954) Using all the QLQ-C30 domains and age as predictors.
Perwitasari et al. [13]	Breast cancer, nasopharyngeal cancer, and colorectal cancer	5L	300	- OLS regression	Full and reduced model showed similar performances. (MAE 0.125, RMSE 0.168).
Wojciechowski et al. [14]	Paroxysmal nocturnal hemoglobinuria, multicenter	5L	71	- OLS regression - ALDVMM - Generalised additive model	OLS regression using all domains without interactions and age, sex as covariate (RMSE 0.0862).

ALDVMM: Adjusted limited dependent variable mixture model; EF: Emotional functioning; FA: Fatigue; MAE: Mean absolute error; OLS: Ordinary least-squares; PF: Physical functioning; PA: Pain; QL: Global health status; RF: Role functioning; RMSE: Root mean squared error; SL: Insomnia

Although earlier studies have developed mapping algorithms for breast cancer patients [3, 10] cultural factors, patient characteristics, and the EQ-5D-5L value sets used in many countries [15] may limit the applicability of existing mapping algorithms to a German breast cancer population. Furthermore, compared with those of patients with early-stage disease, the health-related quality of life (HRQoL) and health utility of patients with metastatic breast cancer are unique challenges.

While the QLQ-C30 is one of the most widely used cancer-specific HRQoL questionnaires, the EORTC Computerized Adaptive Testing Core Item Banks (EORTC CAT Core) is an advanced measurement system designed to assess HRQoL in cancer patients. It is an adaptive version of the QLQ-C30 and enhances measurement precision by dynamically selecting the most relevant questions based on the patient’s responses. This approach reduces respondent burden while maintaining accuracy. Importantly, the EORTC CAT Core maintains conceptual equivalence with the QLQ-C30, ensuring consistency across studies that use either instrument.

Mapping the EORTC CAT Core to EQ-5D-5L is crucial for health economic evaluations. Many clinical trials and real-world studies rely on the EORTC CAT Core or QLQ-C30 for HRQoL data, but often lack direct EQ-5D-5L measurements. Therefore, mapping allows researchers to estimate EQ-5D-5L utility values, making these data more valuable for economic analysis. Moreover, a robust mapping algorithm enables researchers to retrospectively estimate EQ-5D-5L scores, avoiding the need for additional data collection.

To date, there is no validated mapping algorithm for the German EQ-5D-5L in patients with metastatic breast cancer, nor is there a mapping for the EORTC CAT Core to the EQ-5D-5L index. Therefore, the aims of the present study are to develop and compare direct and indirect mapping algorithms from the EORTC QLQ-C30 and CAT Core to the EQ-5D-5L index for the German-based populations.

## Methods

### Data

The data used in this study were collected from the PRO B study [16]. The PRO B study was a multicenter, randomized controlled health service research trial conducted between May 2021 and February 2024. The study enrolled 924 patients who were recruited from 52 medical centers across Germany. Eligible patients were female, over 18 years of age, able to read and understand German, receiving anticancer drug treatment for metastatic breast cancer, and had a life expectancy of more than 3 months at enrollment. Additional eligibility criteria included internet access via a mobile phone and an Eastern Cooperative Oncology Group (ECOG) performance status of 0 to 2. Patients were stratified and randomly assigned at a 1:1 ratio on the basis of study center, site of metastasis, and clinical subtype. They were then allocated to either the intervention group, which received application-based weekly PRO monitoring with alert generation in case of worsening PRO values—followed by physician contact and individualized treatment adaptation—or to a control group that completed a PRO survey every three months without alert generation [16]. Patients in the intervention group completed different short forms from the EORTC CAT item banks [17] weekly, whereas those in the control group completed them every three months. The EQ-5D-5L was assessed at baseline, 6 months, and 12 months via the mobile phone application in both groups. German value sets were used to calculate the utility index [18]. Observations with entirely missing data on either the EORTC items or the EQ-5D-5L were excluded from the analysis. No missing data at the item level were observed, as the PRO B study design did not allow patients to skip PRO items. Therefore, missing data are based on complete and reliable data without the need for imputation.

### Dataset

**Fig. 1:**
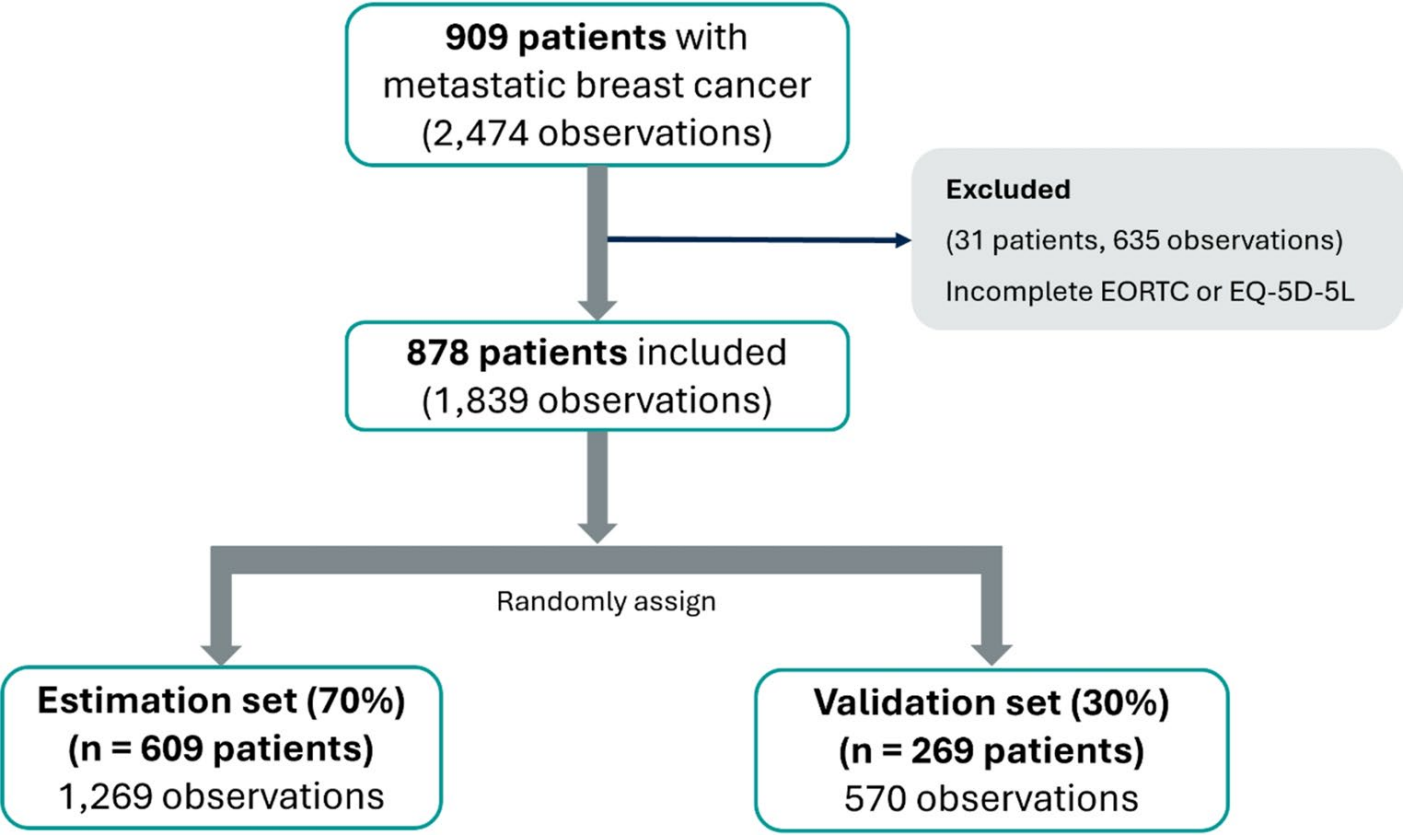
Flowchart

The initial dataset contained 2,474 observations from 909 patients (15 patients were excluded from the PRO-B study because they provided no complete response to any questionnaires). After excluding 635 observations with missing data on the EORTC items or the EQ-5D-5L, the dataset contained 1,839 observations from 878 patients (Fig. 1). Data from the EORTC and the EQ-5D-5L at baseline, 6 months, and 12 months were pooled, and the clustering of multiple responses per patient was adjusted in the mapping models. Since mapping models often perform well on the same data used for their development, validation with external datasets is needed to evaluate actual mapping performance and to avoid overfitting. However, the external datasets are not available in our study. Thus, we used an internal validation sample by splitting the patients in this study into two sets via computer-generated random numbers: 70% of the patients (1,269 observations, 609 patients) were randomly assigned to the ***estimation set*** to generate the mapping model, whereas the remaining 30% (570 observations, 269 patients) constituted the ***validation set*** to test model performance. To ensure similar distributions of disease severity between the estimation and validation sets, we grouped the EQ-5D-5L utility index values for each patient into four quartile-based categories (< 0.775, 0.775–0.876, 0.877–0.942, ≥ 0.943) across all time points. For example, if patient A had real utility index values of 0.760, 0.913, and 0.861 at baseline, 6 months, and 12 months, respectively, we assigned these values to utility groups 1, 3, and 2, respectively. We then stratified patients on the basis of the specific pattern of their utility groupings (e.g., 1, 2, 3 or 3, 2, 1, which are considered equivalent), ensuring that the sequence of the groups was preserved while distinguishing it from a different grouping such as 1, 2, or 4. Finally, we stratified randomized patients according to these pattern groups into either the estimation or validation datasets, ensuring balanced and representative distributions in both sets.

### Instruments

We conducted the EQ-5D-5L assessment via the German version of the EQ-5D-5L questionnaire [15] which includes five items: mobility, self-care, usual activities, pain/discomfort, and anxiety/depression. In the EQ-5D-5L, patients rate their health status on a scale from 1 (no problem) to 5 (extreme problems) for each of these five dimensions. We converted the responses from the EQ-5D-5L into a utility index via the German value set [18] which translates responses into a single index value representing overall health status while accounting for societal and country-specific considerations of HRQoL differences. The German value set provides a utility index ranging from − 0.661 to 1, where 0 represents a health state equivalent to being dead, 1 indicates full health, and values below 0 represent health states that might be considered worse than dead.

The QLQ-C30 (version 3.0) is a standardized, cancer-specific questionnaire developed by the EORTC to assess HRQoL in cancer patients. It includes 30 items covering functional health, symptoms, and overall QoL [19]. In this study, HRQoL was assessed via several short forms from the EORTC CAT Core item banks [17] which were specifically developed for the PRO B study. These short forms served as the source measures in the mapping algorithms and consisted of 51–73 items, with 2–8 items per domain. While the QLQ-C30 consists of 30 items assessing nine multi-item domains, all functional health and symptom domains of the QLQ-C30 are included in the EORTC CAT Core item banks. Both the EORTC CAT Core and the QLQ-C30 encompass five functional scales (physical, role, cognitive, emotional, and social functioning), nine symptom scales (fatigue, pain, nausea/vomiting, dyspnea, insomnia, appetite loss, constipation, diarrhea, and financial difficulties), and a global health status/QoL (GH/QoL) scale. The EORTC CAT Core scores use standardized *T* scores, which are based on a normative metric from a general population with a fixed mean of 50 points and a standard deviation of 10 points [20]. On the other hand, the QLQ-C30 scores are calculated by averaging the items within each scale and then transforming them to a range of 0-100 [21]. High values on a functional scale represent good functioning, whereas high values on a symptom scale or item represent a high symptom burden.

### Statistical analysis

All the statistical analyses were conducted via Stata 18/MP (StataCorp, 2023, College Station, TX, USA). Baseline characteristics are presented via descriptive statistics, including the mean, standard deviation (SD), minimum, maximum, and percentage, as appropriate, separately for the estimation and validation sets. We explored the relationship between each domain of the EORTC CAT Core, QLQ-C30 and EQ-5D-5L via the repeated measures correlation coefficient (r_rm_), which accounts for interindividual variability, as implemented in the R package “*rmcorr*” [22]. 

We followed the ISPOR good practice guidelines for mapping generic health status from nonpreference-based instruments [23] and reported the results following the methodological guidance recommended by the Mapping onto Preference-Based Measures Reporting Standards checklist [24] (Supplementary Tables S1 and S2).

For all direct and indirect mapping models, we performed a full model that included all domains of the EORTC as independent variables. For direct mapping, the dependent variable was the EQ-5D-5L utility index, and we employed four regression types on the basis of previous studies: OLS, Tobit, adjusted beta regression, and ALDVMM. In line with prior EQ-5D mapping studies, the OLS model assumes that the EQ-5D-5L utility index can be estimated as a linear combination of responses from the EORTC questionnaire. Some studies have demonstrated that OLS performs best when the QLQ-C30 is mapped to EQ-5D index values [3, 10, 13, 14]. However, this model may produce inaccurate estimates because of ceiling effects in the EQ-5D index values [25]. To address this bounded utility issue, we applied both the Tobit model and ALDVMM, setting the lower and upper bounds to −0.661 and 1, respectively. ALDVMM offers flexibility in modeling bounded, skewed, and multimodal distributions of health utilities by expressing them as a mixture of continuous distributions [26]. Each observation has a probability of contributing to each component, allowing the model to flexibly approximate complex utility distributions. To identify the global maximum of the likelihood function in the ALDVMM, we used both local and global optimization approaches to improve the likelihood of convergence to the global optimum. First, we fitted a constant-only model and used its estimated parameters to initialize the full model using the “*inimethod(cons)*” option in *aldvmm* [23] command. In addition, we employed a global optimization technique using a simulated annealing, with different starting values and random seeds to broadly explore the parameter space. We then compared the results of both methods using log-likelihood and Bayesian Information Criterion (BIC). In our study, local optimization consistently produced higher likelihoods and better model fit (based on BIC) than global optimization, suggesting that our local optimization method effectively identified a suitable, possible global, solution. We explored models with up to five components. However, the four- and five-component models failed to converge, even after adjusting optimization setting and increasing iterations. Based on convergence, interpretability, and BIC, we retained the one-, two-, and three-component models for further analysis. For the adjusted beta regression, which requires scores between 0 and 1, we addressed the issue of the EQ-5D-5L utility index potentially being negative by transforming it to a 0–1 scale. This transformation was performed via the following formula: (observed utility - (−0.661))/(1 - (−0.661)), where − 0.661 represents the lowest possible utility index in the German value set [18]. The predicted utility index were truncated at one if they were higher than one.

For indirect mapping, we performed generalized ordered logit (GOLOGIT) models for each EQ-5D-5L item (as an ordinal dependent variable) via the Stata command “*gologit2*” [27]. The estimated responses were then combined, and utility values were calculated via the German value set [18]. As each item was modeled separately, each mapping algorithm consisted of five separate models. Given the ordinal scaling of the dependent variable, ordinal logistic regression (OLOGIT) was used to estimate the probability of each response category. However, OLOGIT’s reliance on the proportional odds assumption might be violated; therefore, we opted for the GOLOGIT model, which relaxes the proportional odds assumption, providing a more robust approach for analyzing ordinal data [27, 28].

### Model validation and predictive ability

We report measurements of overall model fit, including the mean absolute error (MAE), root mean squared error (RMSE), mean prediction bias (observed mean - predicted mean), and Lin’s concordance correlation coefficient (CCC). We also present density distribution functions to compare the actual and estimated data, assessing model fit across the distribution of possible values. To evaluate potential systematic bias between the actual and estimated utilities, we used Bland‒Altman plots [29] which display the difference between the observed and estimated utility index values against the average of these values. Additionally, we included LOWESS smoothing with a 95% confidence interval. To assess the consistency of our mapping algorithm across different utility index ranges, we examined its predictive accuracy over the entire range of EQ-5D-5L utility index values, with a particular focus on the intervals < 0.60, 0.60–0.79, and 0.80-1.00.

## Results

**Fig. 2:**
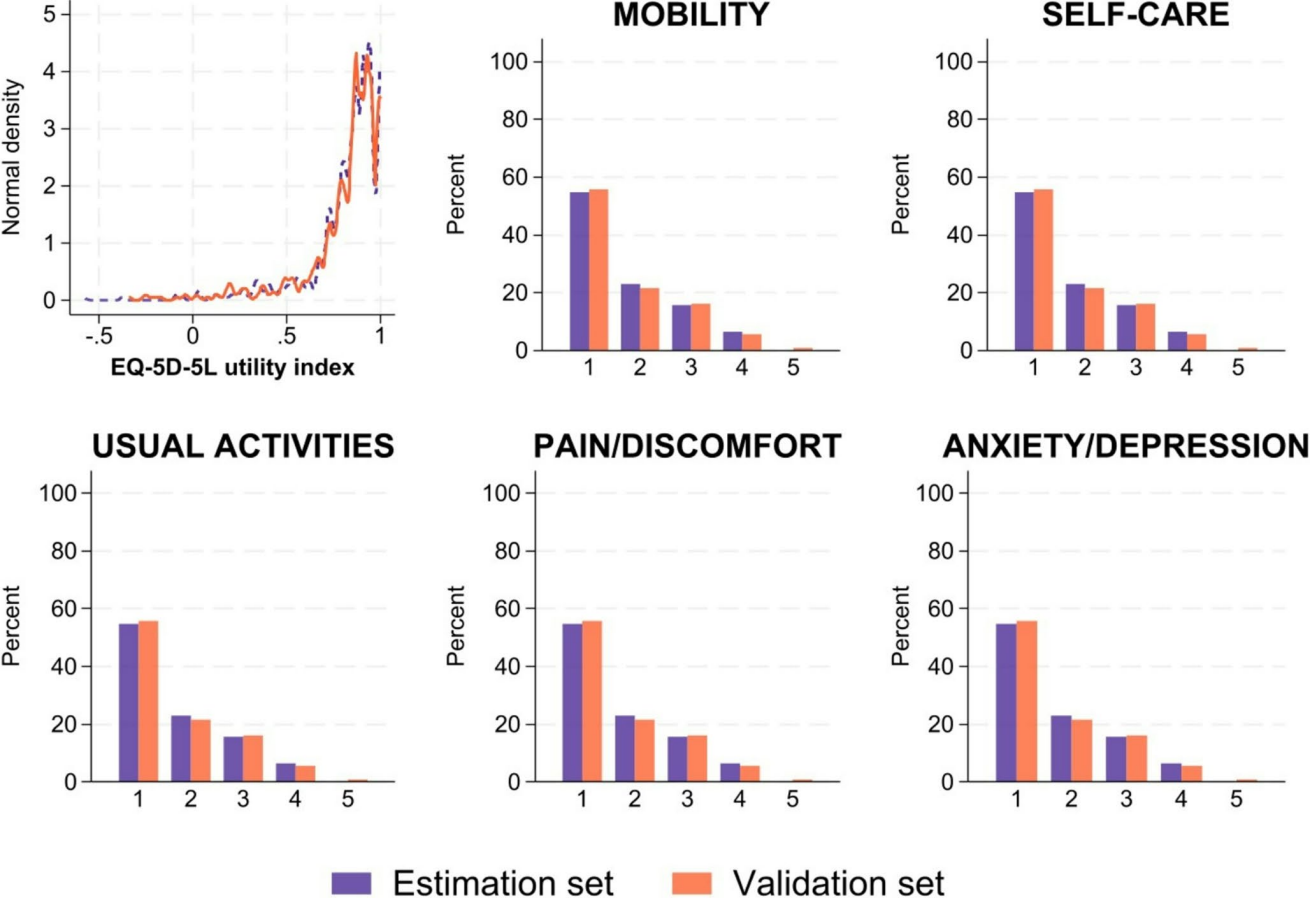
Distributions of the EQ-5D-5L utility index values and health status across the five domains for both the estimation and validation datasets

Overall, the baseline characteristics were similar between the estimation and validation sets (Table 2). The mean age at enrollment was 51 (range 19–83) years. Over 80% of the patients had hormone receptor-positive (HR+) disease, and 50% of the patients had brain or multiple metastases. The observed EQ-5D-5L utility index was left-skewed, ranging between − 0.58 and 1.00 (the estimation set: −0.58 to 1.00 and the validation set: −0.34 to 1.00), and the distribution of each domain level is presented in Fig. 2. The mean EQ-5D-5L utility index was 0.817 (SD 0.204), 11.6% (214/1,839) had a utility index score equal to 1 (full health), and 1.1% (20/1,839) had a utility index score ≤ 0 (poor health state). The mean observed utility indices were 0.819 (SD 0.120) and 0.812 (SD 0.215) for the estimation and validation sets, respectively. The scores of the QLQ-C30 (ranging from 0 to 100) were similarly distributed in both datasets (Table 3).

**Table 2:** Baseline characteristics by estimation and validation datasets

Variables	Estimation dataset	Validation dataset
Number of patients	*n* = 609	*n* = 269
Age at enrolment (years)		
Mean (SD) [Min, Max]	51.0 (10.7) [19, 83]	50.6 (10.9) [23, 83]
<30	10 (1.6%)	8 (3.0%)
30–39	79 (13.0%)	34 (12.6%)
40–49	181 (29.7%)	85 (31.6%)
50–59	211 (34.6%)	87 (32.3%)
60–69	97 (15.9%)	46 (17.1%)
≥70	31 (5.1%)	9 (3.3%)
Histological findings		
HR+/HER- or HR+/HER+	494 (81.1%)	211 (78.4%)
HR-/HER+ or HR-/HER-	115 (18.9%)	58 (21.6%)
Types of distant metastasis		
Brain or multiple	346 (56.8%)	144 (53.5%)
Bone or lymph node or skin	175 (28.7%)	85 (31.6%)
Visceral (only one organ)	88 (14.4%)	40 (14.9%)
Family status	*n* = 600	*n* = 268
Single	71 (11.8%)	44 (16.4%)
Married	386 (64.3%)	179 (66.8%)
Separated/divorced/widowed	143 (23.8%)	45 (16.8%)
Education	*n* = 600	*n* = 268
Low	69 (11.5%)	26 (9.7%)
Medium	324 (54.0%)	148 (55.2%)
High	207 (34.5%)	94 (35.1%)
Health status at baseline	*n* = 601	*n* = 268
Normal, unrestricted activity	120 (20.0%)	56 (20.9%)
Limitation in physical exertion	338 (56.2%)	143 (53.4%)
Able to walk, self-care possible but unable to work	107 (17.8%)	59 (22.0%)
Limited self-care possible	34 (5.7%)	10 (3.7%)
Totally dependent on care	2 (0.3%)	0 (0.0%)

**Table 3:** Distributions of EQ-5D-5L utility index, EORTC QLQ-C30, and EORTC CAT Core scores by estimation and validation dataset

	Estimation dataset (*n*_patient_ = 609, *n*_observation_ = 1,269)		Validation dataset (*n*_patient_ = 269, *n*_observation_ = 570)	
	Mean (SD)	Median (IQR)	Mean (SD)	Median (IQR)
EQ-5D-5L				
Utility index*	0.819 (0.120)	0.877 (0.779–0.943)	0.812 (0.214)	0.877 (0.772–0.943)
VAS	65.5 (20.6)	69.0 (50.0–81.0)	65.9 (20.2)	69.0 (49.0–82.0)
EORTC QLQ C-30				
Global heath/QoL	60.3 (20.4)	66.7 (50.0–75.0)	60.6 (19.6)	66.7 (50.0–75.0)
Physical functioning	71.3 (22.9)	73.3 (53.3–93.3)	71.0 (24.4)	80.0 (53.3–86.7)
Role functioning	75.3 (23.5)	83.3 (66.7–100.0)	76.4 (24.3)	83.3 (66.7–100.0)
Emotional functioning	60.9 (25.7)	66.7 (41.7–83.3)	60.9 (26.6)	66.7 (41.7–83.3)
Cognitive functioning	74.2 (25.5)	83.3 (66.7–100.0)	74.4 (25.2)	83.3 (66.7–100.0)
Social functioning	65.3 (29.4)	66.7 (50.0-100.0)	67.4 (29.9)	66.7 (50.0-100.0)
Fatigue	46.8 (28.4)	44.4 (22.2–66.7)	45.6 (27.9)	44.4 (22.2–66.7)
Nausea and vomitting	10.6 (19.3)	0.0 (0.0-16.7)	10.5 (17.7)	0.0 (0.0-16.7)
Pain	33.6 (29.6)	33.3 (0.0–50.0)	32.3 (29.7)	33.3 (0.0–50.0)
Dyspnea	31.2 (31.7)	33.3 (0.0-66.7)	29.0 (32.2)	33.3 (0.0-33.3)
Insomnia	41.1 (32.9)	33.3 (0.0-66.7)	36.8 (30.9)	33.3 (0.0-66.7)
Appetite loss	18.8 (28.8)	0.0 (0.0-33.3)	18.2 (28.0)	0.0 (0.0-33.3)
Constipation	18.5 (28.5)	0.0 (0.0-33.3)	16.1 (27.0)	0.0 (0.0-33.3)
Diarrhea	21.5 (30.1)	0.0 (0.0-33.3)	20.7 (27.7)	0.0 (0.0-33.3)
Financial difficulties	19.1 (29.7)	0.0 (0.0-33.3)	19.6 (29.2)	0.0 (0.0-33.3)
EORTC CAT Core ( ***T*** -score)				
Global heath/QoL	36.1 (4.0)	36.0 (34.8–36.7)	35.8 (3.4)	36.0 (34.8–36.7)
Physical functioning	42.2 (9.8)	41.8 (35.5–47.8)	42.5 (10.3)	43.0 (35.5–48.3)
Role functioning	41.0 (10.1)	40.0 (34.3–47.3)	41.4 (10.3)	40.5 (34.2–47.6)
Emotional functioning	44.0 (9.4)	43.1 (37.1–50.9)	44.1 (9.7)	43.2 (37.3–51.8)
Cognitive functioning	44.6 (10.2)	44.0 (38.4–57.3)	45.0 (10.0)	44.5 (38.4–57.3)
Social functioning	42.8 (9.5)	41.8 (36.0-50.3)	43.4 (9.7)	42.5 (36.5–51.3)
Fatigue	56.8 (10.0)	56.1 (50.4–63.7)	56.4 (9.9)	56.1 (49.9–62.7)
Nausea and vomitting	58.1 (10.2)	51.7 (51.7–64.4)	58.2 (9.7)	51.7 (51.7–64.4)
Pain	53.7 (9.9)	55.6 (41.4–60.3)	52.9 (9.9)	54.3 (41.4–60.3)
Dyspnea	57.2 (10.7)	59.3 (42.5–67.2)	56.5 (10.8)	59.3 (42.5–63.7)
Insomnia	56.3 (8.8)	55.3 (49.9–63.0)	55.0 (8.4)	55.3 (49.9–63.0)
Appetite loss	53.7 (11.1)	45.9 (45.9–64.6)	53.7 (10.9)	45.9 (45.9–64.6)
Constipation	52.1 (10.5)	43.6 (43.6–59.7)	51.0 (10.0)	43.6 (43.6–59.7)
Diarrhea	54.2 (11.7)	45.2 (45.2–64.2)	54.2 (11.0)	45.2 (45.2–64.2)
Financial difficulties	53.5 (10.4)	46.1 (46.1–63.1)	53.6 (10.1)	46.1 (46.1–63.1)

*the utility index is based on German value set

The relationships between the EORTC domains and the EQ-5D-5L were mostly low to moderate. Moderate correlations were observed for GH/QoL, physical functioning, emotional functioning, social functioning, fatigue, and pain (r_rm_ >0.3) (Supplementary Table S3).

### Direct mapping

**Fig. 3:**
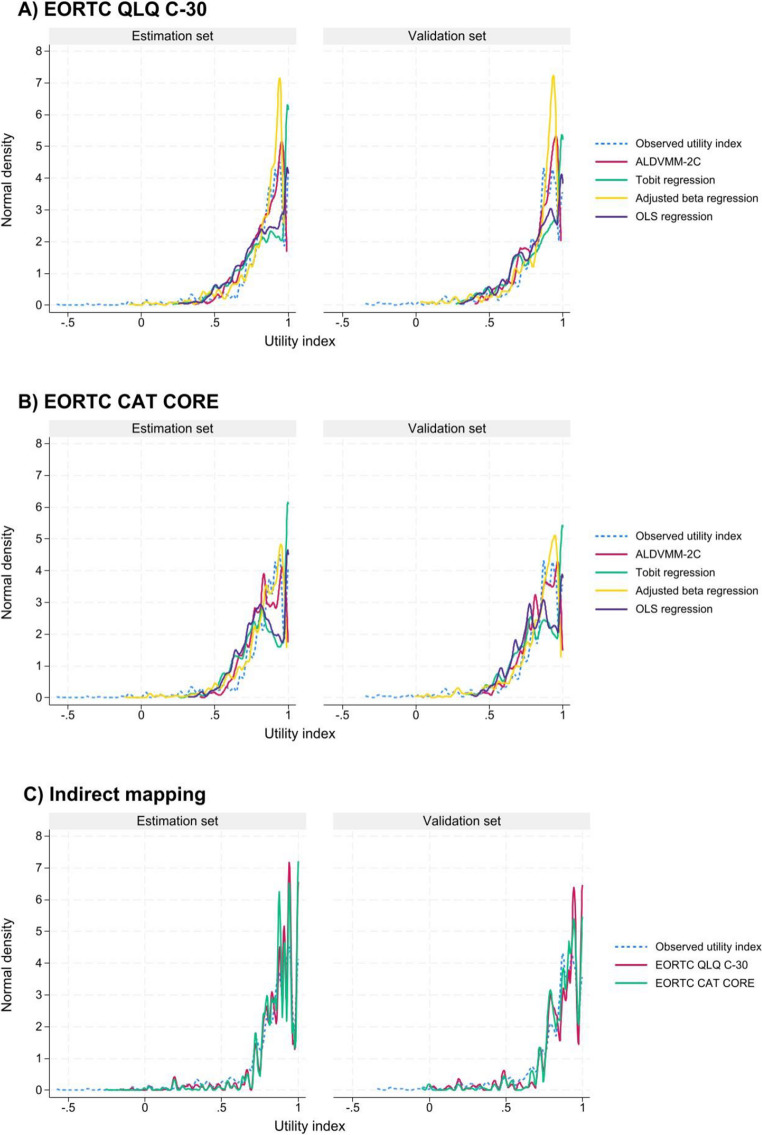
Density distributions between observed and predicted utility index values from each model. ALDVMM = adjusted limited dependent variable mixture models; OLS, ordinary least squares

**Fig. 4:**
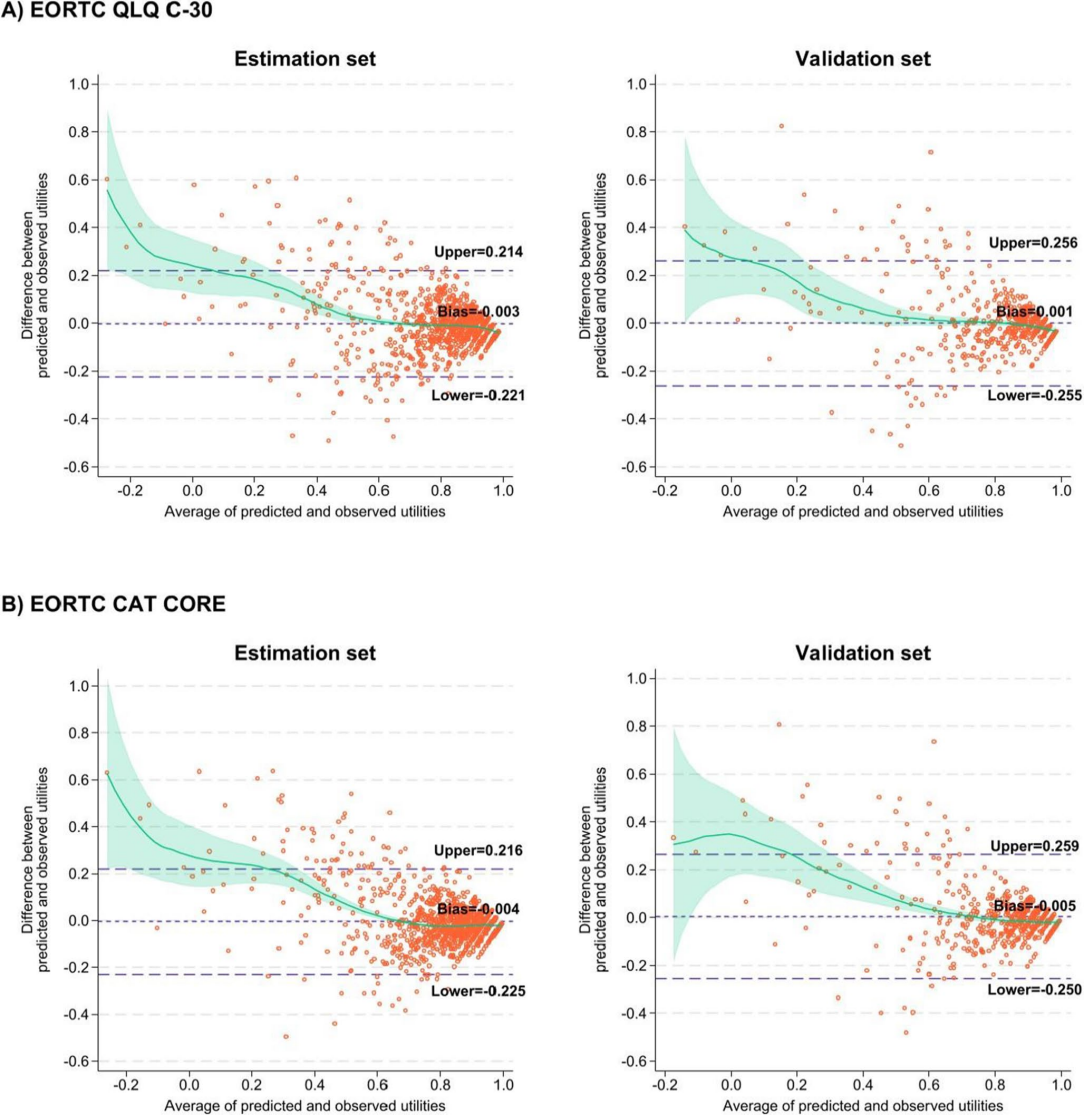
Bland-Altman plot of the observed and predicted mean differences utility index values of the direct mapping using the adjusted beta regression model. The light green line represents a LOWESS smoothing with a 95% confidence interval

Table 4 presents the performance statistics for the estimation and validation sets of the direct and indirect mapping algorithms. The adjusted beta regression was the best-performing model for direct mapping in both the CAT Core and the QLQ-C30 (Supplementary Sect. 1 and Table S4 present the mapping algorithm of the adjusted beta regression). The estimated mean utility index values were close to the observed values with both the estimation and validation sets (the predicted mean utility indices were as follows: QLQ-C30 = 0.815 and 0.813; CAT Core = 0.815 and 0.817). Compared with those of the other models, the measures of error (MAE and RMSE) were the lowest. Moreover, this model showed the highest CCC between the observed and estimated utilities in both datasets (QLQ-C30: estimation 0.817 and validation 0.781; CAT Core: estimation 0.804 and validation 0.770), and the mean prediction bias was close to zero (−0.004). Figure 3 shows the distributions of observed and estimated utility index values from each model, whereas Supplementary Figure S1 displays these distributions specifically for the ALDVMM-1C, 2C, and 3C models. The density of the distributions shows good overlap across the distribution. However, in the adjusted beta regression model using the QLQ-C30 for mapping (Fig. 3A), there is a relatively high, flat section with no noticeable peak. In contrast, the CAT Core shows good overlap with more consistent distribution shapes in both the estimation and validation sets (Fig. 3B). Moreover, the Bland‒Altman plot revealed that the adjusted beta regression overestimated when the utility index values were less than 0.6 (Fig. 4). OLS regression also provided good results, with a mean prediction bias of zero. However, the OLS regression assumes the normality of the residuals, and this assumption is violated in this analysis. Therefore, the linear regression estimated utilities outside of the plausible range of the EQ-5D-5L utility index values (i.e., utility value > 1). The Tobit model similarly estimated utility index values greater than one and had the highest MAE (0.09–0.10), RMSE (0.13–0.14), and mean prediction bias (0.014–0.017) for both the QLQ-C30 and the CAT Core.

**Table 4:** Predictive performances of the direct and indirect mapping algorithms

	Estimation set (*n*_patient_ = 609, *n*_observation_ = 1,269) Mean observed utilities = 0.819 (SD 0.120)					Validation set (*n*_patient_ = 269, *n*_observation_ = 570) Mean observed utilities = 0.812 (SD 0.215)				
	MAE	RMSE	Mean predict	Mean prediction bias	CCC	MAE	RMSE	Mean predict	Mean prediction bias	CCC
EORTC QLQ C-30										
Direct mapping										
ALDVMM-1C	0.079	0.119	0.809	−0.010	0.772	0.088	0.137	0.810	−0.003	0.733
ALDVMM-2C	0.075	0.126	0.832	0.013	0.713	0.084	0.144	0.833	0.021	0.672
ALDVMM-3C	0.076	0.126	0.831	0.012	0.715	0.084	0.143	0.832	0.020	0.673
Tobit regression	0.081	0.121	0.825	0.005	0.778	0.090	0.139	0.826	0.014	0.740
* Adjusted beta regression*	*0.072*	*0.111*	*0.815*	*−0.004*	*0.817*	*0.081*	*0.130*	*0.813*	*0.001*	*0.781*
OLS regression	0.080	0.122	0.816	−0.003	0.763	0.090	0.140	0.817	0.005	0.723
Indirect mapping										
GOLOGIT	0.074	0.127	0.855	0.035	0.761	0.080	0.140	0.853	0.041	0.748
EORTC CAT Core ( ***T*** -score)										
Direct mapping										
ALDVMM-1C	0.084	0.126	0.806	−0.013	0.732	0.089	0.141	0.811	−0.001	0.694
ALDVMM-2C	0.078	0.133	0.835	0.015	0.667	0.084	0.149	0.838	0.026	0.627
ALDVMM-3C	0.078	0.134	0.835	0.016	0.658	0.085	0.150	0.839	0.027	0.620
Tobit regression	0.083	0.126	0.820	0.001	0.743	0.090	0.143	0.826	0.014	0.705
*Adjusted beta regression*	*0.074*	*0.113*	*0.815*	*−0.004*	*0.804*	*0.081*	*0.130*	*0.817*	*0.005*	*0.770*
OLS regression	0.086	0.131	0.813	−0.006	0.708	0.092	0.147	0.817	0.005	0.668
Indirect mapping										
GOLOGIT	0.075	0.132	0.860	0.041	0.727	0.079	0.141	0.860	0.048	0.724

ALDVMM = adjusted limited dependent variable mixture models; CCC = Lin’s concordance correlation coefficient; GOLOGIT = generalized ordered logit models; MAE = mean absolute error; OLS, ordinary least squares; RMSE = root mean standardized error

### Indirect mapping

**Fig. 5:**
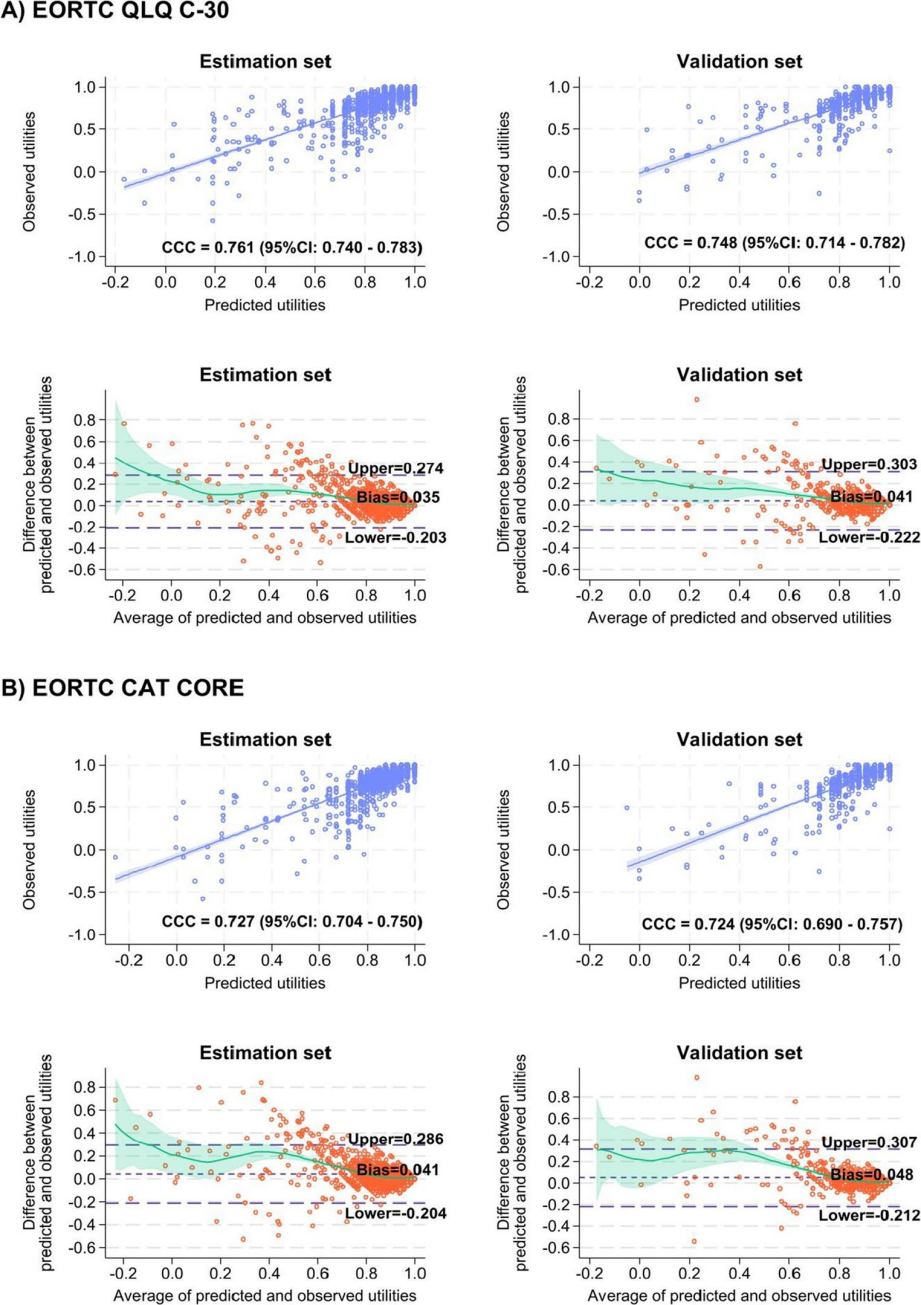
Scatter-plot and Bland-Altman plots showing the observed and predicted utility index values in indirect mapping models. The light green line represents a LOWESS smoothing with a 95% confidence interval. CCC = Lin’s concordance correlation coefficient

The performance of the indirect mapping algorithms for both the QLQ-C30 and the CAT Core using GOLOGIT is presented in Table 4 and in Supplementary Table S5–S6. The indirect mapping algorithms overestimate the utility index in both the EORTC dataset and the datasets (predicted mean utility index for the QLQ-C30: estimation set = 0.855 [a mean prediction bias = 0.035] and validation set = 0.853 [a mean prediction bias = 0.041]; the CAT Core: estimation set = 0.860 [a mean prediction bias = 0.041] and validation set = 0.860 [a mean prediction bias = 0.048]). The MAE and RMSE were approximately 0.08 and 0.13, respectively, for both datasets. The density distributions between the observed and predicted utilities were similar for both the QLQ-C30 and the CAT Core separately in the estimation and validation sets, although the indirect mapping algorithms provided a slightly greater proportion of full health (utility = 1) than did the observed data (Fig. 3C). The 95% limits of agreement were estimated at −0.203 to 0.274 (mean 0.035) for the QLQ-C30 and at −0.204 to 0.286 (mean 0.041) for the CAT Core (Fig. 5A and B). The Bland‒Altman plots indicated that the utility indices of patients with lower utility index values (< 0.6) tended to be overestimated for both EORTCs.

### Assessment of mapping performance across the range of plausible utility scores

The MAEs were calculated across the full range of observed EQ-5D utility index values. For all the mapping algorithms, the MAEs were the smallest for higher observed utility index values but increased considerably when the observed utility index values dropped below 0.6, indicating poor model fit for lower utility index values. Among the direct mapping models, all performed well when the observed utility index values exceeded 0.6, with the adjusted beta regression model demonstrating the best fit, as indicated by the lowest MAEs compared with the other models. The indirect mapping models fit the observed data well when the observed utility index values were above 0.8 for both EORTC measures. However, for the lower values across both the direct and indirect mapping algorithms, the fit statistics were much poorer, with considerably worse fit statistics when the observed utility index values fell below 0.6 (Table 5).

**Table 5:** Mean absolute error across EQ-5D-5L utility index range

	Estimation set (*n*_patient_ = 609, *n*_observation_ = 1,269) Mean observed utilities = 0.819 (SD 0.120)				Validation set (*n*_patient_ = 269, *n*_observation_ = 570) Mean observed utilities = 0.812 (SD 0.215)			
	MAE (Obs. = 1,269)	MAE across utility index range			MAE (*n* = 570)	MAE across utility index range		
		< 0.60 (*n* = 131)	0.60–0.80 (*n* = 255)	0.80–1 (*n* = 883)		< 0.60 (*n* = 65)	0.60–0.80 (*n* = 113)	0.80–1 (*n* = 392)
EORTC QLQ C-30								
Direct mapping								
ALDVMM-1C	0.079	0.218	0.087	0.056	0.088	0.269	0.101	0.054
ALDVMM-2C	0.075	0.277	0.070	0.047	0.084	0.322	0.075	0.046
ALDVMM-3C	0.076	0.275	0.070	0.048	0.084	0.321	0.075	0.047
Tobit regression	0.081	0.218	0.089	0.058	0.090	0.270	0.104	0.057
Adjusted beta regression	*0.072*	*0.201*	*0.095*	*0.047*	*0.081*	*0.242*	*0.111*	*0.046*
OLS regression	0.080	0.226	0.083	0.058	0.090	0.278	0.098	0.057
Indirect mapping								
GOLOGIT	0.074	0.261	0.096	0.040	0.080	0.292	0.103	0.039
EORTC CAT Core ( ***T*** -score)								
Direct mapping								
ALDVMM-1C	0.084	0.243	0.075	0.062	0.089	0.295	0.080	0.057
ALDVMM-2C	0.078	0.309	0.059	0.048	0.084	0.349	0.064	0.046
ALDVMM-3C	0.078	0.315	0.058	0.049	0.085	0.353	0.063	0.046
Tobit regression	0.083	0.244	0.078	0.061	0.090	0.296	0.082	0.058
Adjusted beta regression	*0.074*	*0.219*	*0.083*	*0.049*	*0.081*	*0.263*	*0.097*	*0.046*
OLS regression	0.086	0.259	0.070	0.064	0.092	0.309	0.077	0.061
Indirect mapping								
GOLOGIT	0.075	0.295	0.093	0.037	0.079	0.317	0.089	0.036

ALDVMM = adjusted limited dependent variable mixture models; GOLOGIT = generalized ordered logit models; MAE = mean absolute error; obs. = number of observations; OLS = ordinary least squares

## Discussion

In this study, we developed algorithms for direct and indirect mapping of the EORTC CAT Core and the QLQ-C30 onto the EQ-5D-5L to estimate the utility index in patients with metastatic breast cancer. The adjusted beta regression model was the best-performing model for direct mapping, showing the lowest MAE and RMSE compared to the other models in both the estimation and validation sets. Moreover, the robustness of the indirect mapping model was also observed in our study. In times of increasing financial pressure on healthcare systems, health economic evaluations are essential alongside effectiveness analyses of clinical studies to ensure high-value medical care. These mapping models thus make it possible to translate utility values from the EORTC CAT Core and the QLQ C30, enabling comprehensive economic evaluations for patients with metastatic breast cancer across a variety of healthcare settings.

Our adjusted beta regression model demonstrated the best fit across the validation set for both the EORTC CAT Core and the QLQ-C30 and has also been shown to perform well in mapping to the EQ-5D in previous studies [5–7, 12, 30]. In this study, the MAE was estimated at 0.07, which is acceptable, although it slightly exceeds the recently reported minimal important difference (MID) of 0.06 for the EQ-5D utility index in cancer patients [31]. There are no universally accepted cutoff values for determining whether a model is suitable for practical application. However, the mean prediction bias was minimal, with a mean of −0.004 for both the QLQ-C30 and the CAT Core. Given the small mean prediction bias, this model is likely well suited for assessing cost-effectiveness in patients with metastatic breast cancer. Compared with a study by Kim et al., which mapped the QLQ-C30 and the QLQ-BR23 onto the EQ-5D-5L in metastatic breast cancer patients in Korea via an OLS model incorporating all the QLQ-C30 domains [3] our adjusted beta regression model and the OLS models demonstrated better performance in terms of prediction accuracy, including less bias and a smaller MAE. Although patient demographics, such as age, ECOG status, and mean utility index values, were similar across the studies, the larger sample size in our study, along with our method of randomly assigning samples, could explain the improved accuracy.

In selecting models, we decided to use all domains of the EORTC (15 domains) as predictors to avoid overfitting to the dataset and maximize prediction accuracy [12] as previous research has indicated that, compared with models that incorporate demographic and clinical variables, only the QLQ-C30 model has better predictive performance for the EQ-5D utility index [3]. Additionally, to ensure the practical applicability of the mapping algorithm across diverse patient samples, we chose to not include any sociodemographic variables in the mapping model. Previous studies have reduced mapping models by using selected domains of the EORTC, commonly with the domains of quality of life; physical, emotional and role functioning; and pain [6, 7, 13]. However, the value of adding additional EORTC domains to mapping models has remained uncertain, as there has been no conclusive evidence and the associations with health states vary across different disease entities. Despite this, quality of life, functional domains, and some symptom domains, such as pain and fatigue, are likely to have a significant impact on HRQoL in metastatic breast cancer patients. Therefore, we argue that they should be prioritized when calculating utility index values for health economic evaluations. Moreover, our mapping algorithms for the QLQ-C30 were developed using all the observed items of the QLQ-C30. However, in real-world settings, incomplete questionnaires with missing items are common. In such cases, we recommend considering the algorithm for the EORTC CAT Core, which is based on item response theory and can better accommodate missing item data.

Many studies have used OLS models to map the EORTC QLQ-C30 to the EQ-5D [3, 8, 10, 13, 14] despite the well-known limitation of OLS models, which produce invalid upper-range utility estimates (i.e., utility index values > 1). In our study, while OLS models exhibited the lowest bias in both the estimation and validation sets for both EORTC measures, we also encountered the issue of predicted values exceeding one. This issue, however, can be addressed by truncating the predicted utilities to the upper boundary value [3] and including a square of the EORTC scores for dealing with nonlinearity effects could improve the prediction of the OLS model [23]. Although recent guidelines recommend beta-based regression for mapping models over OLS, few studies have adopted this approach [7, 9, 12, 14]. While beta regression has shown slightly better predictive accuracy, it is more complex in external studies because of the need to transform utility estimates to fit within the 0–1 range [30].

We noticed similar problems in developing mapping algorithms to predict the EQ-5D utilities from the EORTC CAT Core and QLQ-C30 regarding counterintuitive signs for the regression coefficients of the EORTC domains [3]. For example, the regression coefficient for social functioning has a negative sign, which counterintuitively suggests a worse QoL when social functioning improves. Similarly, some symptom domains, such as fatigue, dyspnea, constipation, and diarrhea, had positive coefficients, implying an improved QoL when these symptoms worsened. These unexpected signs could be due to several factors, including high correlation among EORTC domains (multicollinearity), model misspecification (such as including irrelevant or omitting key variables), overfitting, or boundary effects of the EQ-5D. If multicollinearity or overfitting contributes to these counterintuitive results, simplifying the model or using techniques such as principal component analysis could help resolve these issues and improve the model’s performance.

In this analysis, overpredictions at poorer health states are also presented for both direct and indirect mapping models. Similar issues were observed in previous publications regarding mapping [10, 12, 13, 32]. The reasons for overestimation may be due to several factors, including the functional form of the model, the range of the scale, and the number of health states [5]. On the other hand, the number of observations in the published datasets (including those in our study) for low utility index values is generally low, which may impact the prediction fit and regression estimates [23]. Our findings contrast with those suggesting that using ALDVMM could overcome this issue. For example, studies by Gray et al., which mapped the QLQ-C30 onto the EQ-5D-3L in HER2-positive advanced breast cancer patients, and Wojciechowski et al., which mapped the QLQ-C30 onto the EQ-5D-5L for patients with paroxysmal nocturnal hemoglobinuria, reported that the ALDVMM outperformed other models for both patients with very good and very poor health [11, 14]. The choice of mapping algorithm often depends on disease severity, with linear models being more suitable for patients in good health, whereas beta and mixture models tend to perform better for those with poorer health [6, 11].

The indirect mapping model performed well and comparably to the preferred direct mapping algorithm and can be applied using any EQ-5D-5L national value set in both EORTC measurements. Similar performance results were reported in another study [12]. However, several limitations of indirect mapping could arise, including loss of precision, limited ability to capture specific health dimensions, reduced accuracy at extreme utility values, complexity and potential for overfitting, lack of universally accepted methods, and bias in health states not covered by the QLQ-C30. The two-step process can introduce errors, leading to less accurate predictions. The QLQ-C30 may not capture all health states relevant to the EQ-5D, particularly outside the population of patients with metastasized breast cancer.

In our study, we estimated the QLU-C10D utility index using the German value set [33] and observed a mean of 0.642 (SD 0.252), with 3.8% of patients (51/1,839) reporting full health and 0.2% (3/1,839) in the poorest health state. In contrast, utility scores derived from mapping EQ-5D-5L using the same QLQ-C30 data yielded a higher mean of 0.815 (SD 0.170). This discrepancy suggests that the QLU-C10D, which includes cancer-specific dimensions such as fatigue, pain, and emotional functioning, may capture a broader or more severe perception of disease burden in metastatic breast cancer patients [34, 35]. The CCC between two utilities was 0.527 (*r* = 0.695) indicates only moderate agreement. This suggests that while the two instruments show some consistency, they are not interchangeable and might capture different aspects of HRQoL [35]. Given the substantial mean difference in utilities, these findings have important implications for cost-effectiveness analyses, as QALY estimates can vary depending on the utility measure used. Further research is warranted to examine the impact of these differences on health economic evaluations in metastatic breast cancer and to guide appropriate utility measure selection in this population.

Other limitations to our study must be considered. First, only data from a clinical trial setting were used. The trial population may not fully represent the real-world clinical setting because a more homogenous study population results from the inclusion and exclusion criteria of the RCT. We used multiple records per patient to increase our sample size for fitting the mapping models, accounting for the potential correlation within patients, assuming that the correlation between the EORTC and the EQ-5D-5 L remains stable over time. Although our sample size was considerably larger than that in many previous mapping studies, the variation in utility index values below one was somewhat limited, which may have affected the precision of the estimates at lower utility levels. To address this, we explored a range of potential mapping models as part of the study. Additionally, we acknowledge that our use of an internal validation dataset limits the generalizability of the model to external populations. While internal validation provides some insight into model performance, it does not fully address the potential differences in external datasets. This approach is a limitation and future validation using independent external datasets with differing characteristics is essential to fully assess model transportability. Moreover, internal validation reduced the sample size available for developing the mapping models, which likely impacted the performance of the ALDVMMs. Since these models require larger sample sizes to estimate a greater number of parameters, this reduction may explain their underperformance. Since there is no gold standard approach for selecting the best mapping algorithms in the literature, we assessed model performance using multiple criteria, including error statistics (e.g., MAE, RMSE), internal validation, mean prediction bias, and CCC. These were evaluated both for the full sample and across different health status levels to ensure predictive robustness. In addition to these statistical metrics, we also considered logical consistency as an essential criterion. Logical consistency refers to the expectation that predicted utility values should align with theoretical and clinical expectations, for example, more severe health states should not be assigned higher utility values than less severe ones. While numerical performance metrics assess statistical fit, they do not always capture implausible or counterintuitive predictions. Therefore, we manually reviewed the model outputs by examining the distribution of predicted utilities and using Bland-Altman plots to assess agreement. This helped ensure that predictions were coherent with the known structure of the EQ-5D-5 L utility scale and the clinical severity indicated by QLQ-C30 or CAT Core responses. Incorporating logical consistency as a qualitative check enhances the credibility of the model and helps avoid selecting algorithms that may appear optimal numerically but fail to behave reasonably across the health severity spectrum. This reinforces the robustness and interpretability of our selected model.

## Conclusions

This study developed algorithms to map the EORTC CAT Core and the QLQ-C30 onto the EQ-5D-5L for patients with metastatic breast cancer. The robustness and prediction precision of direct mapping via adjusted beta regression confirmed it as a superior mapping approach for both EORTC measures compared with other models for a German-based population. The robustness of the indirect mapping algorithm is shown in the estimated and validated datasets. With this indirect mapping algorithm, the EORTC CAT Core and QLQ-C30 can be translated to quality-adjusted life-years for health economic evaluations in any country tariff.

## Supplementary information

Below is the link to the electronic supplementary material.

ESM 1 (DOCX 5.08 KB)

ESM 2 (DOCX 5.40 KB)

ESM 3 (DOCX 1.58 MB)

## Data Availability

The data and the analysis code used in this study are available from the corresponding author upon reasonable request.

## Abbreviations

ALDVMM: Adjusted limited dependent variable mixture model
CAT: Computer Adaptive Testing
CCC: Concordance correlation coefficient
CI: Confidence interval
ECOG: Eastern Cooperative Oncology Group
EORTC: European Organization for Research and Treatment of Cancer
EQ-5D-5L: EuroQoL 5-Dimension with 5 levels
GCP: Good clinical practice
GOLOGIT: Generalized ordered logit model
HER: Human epidermal growth factor receptor
HR: Hormone receptor
HRQoL: Health-related quality of life
MAE: Mean absolute error
OLS: Ordinal least squares
OLOGIT: Ordinal logistic regression model
PROs: Patient-reported outcomes
PROMs: patient-reported outcome measures
RMSE: Root mean squared error
QALYs: Quality-adjusted life years
QoL: Quality of life
QLU-C10D: Quality of Life Utility—Core 10 Dimensions
SD: Standard deviation
