# Supplementary material for: Translating the EORTC CAT core and the QLQ-C30 to the EQ-5D-5L in patients with metastatic breast cancer: A comparison of direct and indirect mapping algorithms
Source: Eur J Health Econ. 2025 Aug 21;27(2):435–51. doi: 10.1007/s10198-025-01824-0 (PMC13046628; doi:10.1007/s10198-025-01824-0)
Supplement: Supplementary file 3 — (DOCX 1.58 MB) [file 10198_2025_1824_MOESM3_ESM.docx]

**Supplementary materials**

**Translating the EORTC CAT Core and the QLQ-C30 to the EQ-5D-5 L in patients with metastatic breast cancer: A comparison of direct and indirect mapping algorithms**

**Table S1** Checklist of items to include when reporting a mapping study

**From: [The MAPS Reporting Statement for Studies Mapping onto Generic Preference-Based Outcome Measures: Explanation and Elaboration](https://link.springer.com/article/10.1007/s40273-015-0312-9)**

| **Section/topic** | **Item number** | **Recommendation** | **Reported on page number/line number** |
| --- | --- | --- | --- |
| **Title and abstract** | | | |
| Title | 1 | Identify the report as a study mapping between outcome measures. State the source measure(s) and generic, preference-based target measure(s) used in the study | P 1 |
| Abstract | 2 | Provide a structured abstract including, as applicable: objectives; methods, including data sources and their key characteristics, outcome measures used and estimation and validation strategies; results, including indicators of model performance; conclusions; and implications of key findings | P 2-3 |
| **Introduction** | | | |
| Study rationale | 3 | Describe the rationale for the mapping study in the context of the broader evidence base | P 4-5 |
| Study objective | 4 | Specify the research question with reference to the source and target measures used and the disease or population context of the study | P 5 |
| **Methods** | | | |
| Estimation sample | 5 | Describe how the estimation sample was identified, why it was selected, the methods of recruitment and data collection, and its location(s) or setting(s) | P 6 |
| External validation sample | 6 | If an external validation sample was used, the rationale for selection, the methods of recruitment and data collection, and its location(s) or setting(s) should be described | NA |
| Source and target measures | 7 | Describe the source and target measures and the methods by which they were applied in the mapping study | P 5-6 |
| Exploratory data analysis | 8 | Describe the methods used to assess the degree of conceptual overlap between the source and target measures |  |
| Missing data | 9 | State how much data were missing and how missing data were handled in the sample(s) used for the analyses | P 7 line 12-13 |
| Modelling approaches | 10 | Describe and justify the statistical model(s) used to develop the mapping algorithm | P 9-10 |
| Estimation of predicted scores or utilities | 11 | Describe how predicted scores or utilities are estimated for each model specification | P 9-10 |
| Validation methods | 12 | Describe and justify the methods used to validate the mapping algorithm | P 10 |
| Measures of model performance | 13 | State and justify the measure(s) of model performance that determine the choice of the preferred model(s) and describe how these measures were estimated and applied | P 10 |
| **Results** | | | |
| Final sample size(s) | 14 | State the size of the estimation sample and any validation sample(s) used in the analyses (including both number of individuals and number of observations) | P 10 |
| Descriptive information | 15 | Describe the characteristics of individuals in the sample(s) (or refer back to previous publications giving such information). Provide summary scores for source and target measures, and summarise results of analyses used to assess overlap between the source and target measures | P 10  Table 1 – Table 2 |
| Model selection | 16 | State which model(s) is(are) preferred and justify why this(these) model(s) was(were) chosen | P 11-12 |
| Model coefficients | 17 | Provide all model coefficients and standard errors for the selected model(s). Provide clear guidance on how a user can calculate utility scores based on the outputs of the selected model(s) | Supplementary |
| Uncertainty | 18 | Report information that enables users to estimate standard errors around mean utility predictions and individual-level variability | Table 3 |
| Model performance and face validity | 19 | Present results of model performance, such as measures of prediction accuracy and fit statistics for the selected model(s) in a table or in the text. Provide an assessment of face validity of the selected model(s) | Table 3 |
| **Discussion** | | | |
| Comparisons with previous studies | 20 | Report details of previously published studies developing mapping algorithms between the same source and target measures and describe differences between the algorithms, in terms of model performance, predictions and coefficients, if applicable | P13-16 |
| Study limitations | 21 | Outline the potential limitations of the mapping algorithm | P 16-17 |
| Scope of applications | 22 | Outline the clinical and research settings in which the mapping algorithm could be used | P 14 |
| **Other** | | | |
| Additional information | 23 | Describe the source(s) of funding and non-monetary support for the study, and the role of the funder(s) in its design, conduct and report. Report any conflicts of interest surrounding the roles of authors and funders | P 19 |

Table S2 Mapping to Estimate Health-State Utility from Non-Preference-Based Outcome Measures: An ISPOR Good Practices for Outcomes Research Task Force Report. Summary of reporting of mapping studies recommendations

| **Recommendation** | **Reported** |
| --- | --- |
| 1. Describe relevant differences between data sets that are candidates for mapping estimation. | Only one dataset was used, which was collected for the purpose of this mapping study, but the data were split into two estimation and validation sets using computer-generated random numbers. |
| 2. Give full details of the selected data set. Describe how the study was run and patients were sampled. Provide baseline and follow up characteristics including the distribution of patients’ disease severity. Missingness in the longitudinal pattern of responses should be described. | The data used in this study was collected from the PRO B study. The PRO B study was a multicenter, randomized controlled health service research trial conducted between May 2021 and February 2024. The study enrolled 924 patients who were recruited from 52 medical centers across Germany. Patients in the intervention group completed different short forms from the EORTC CAT item banks14 weekly, while those in the control group completed them every 3 months. The EQ-5D-5L was assessed at baseline, 6 months, and 12 months via the mobile phone application in both groups. German value sets were used to calculate the utility index15. Observations with missing data on either the EORTC items or the EQ-5D-5L were excluded from the analysis. The initial data set contained 2,474 observations from 909 patients included in the final PRO B study analysis. After excluding 635 observations with missing data on the EORTC items or the EQ-5D-5L, the data set contained 1,839 observations from 878 patients (Figure 1).  Baseline characteristics were presented in Table 2. |
| 3. Plot the distribution of the utility data. | Distribution of the observed utilities presented in Figure 2.  Distribution of the observed and predicted utilities presented in Figure 3. |
| 4. Justify the type of model(s) selected with reference to the characteristics of the target utility distribution and the proposed use of the mapping function. | The justification of the models selected is presented in page 9. |
| 5. Compare the dimensions of health covered by the target utility instrument and those covered by the explanatory clinical measure(s). | Description of health status is provided in Table 3.  Repeated measure correlation coefficients are presented in Table S1. |
| 6. Describe the approach to determining the final model. Include tests conducted and judgments made. | Described in Page 10 |
| 7. Summary measures of fit are of limited value for the total sample. Provide information on fit conditional on disease severity as measured by the clinical outcome measure(s). A plot of mean predicted versus mean observed utility conditional on the clinical variable(s) should be included. | A range of summary measures are presented in Table 3. Distribution of the observed versus predicted utilities presented in Figure 3. |
| 8. Coefficient values, error term(s) distributions(s), variances, and covariances are required. | Presented in Supplementary Table S2, S3, S4. |
| 9. Provide an example predicted value for some sets of covariates. Consider providing a program that calculates predictions for user-defined inputs. | Example of how to estimate predicted utility value presented in Supplementary. Stata code files are provided for both direct and indirect mapping. |
| 10. Parameter uncertainty in a mapping regression should be reflected using standard methods for PSA. Assessment of model suitability for use in cost-effectiveness analysis should also consider the distribution of utility values for PSA, with particular focus on whether these lie outside the feasible utility range for the PBM. | Table 4 presents the range for predicted utilities by each model. |
| 11. When imputing data from a mapping function, individual-level variability should be incorporated using simulation methods and information about the distribution of the error term(s). These simulated data can be compared with the raw observed data, including an assessment of the range of values compared with the feasible range for the PBM. | Not applicable – no imputation conducted. |
| 12. Re-estimation of mapping results in a separate data set or other forms of validation are not routinely required. | no external dataset was available, and only internal validation was applied in this study (mentioned in Page 6-7). |

**Table S3** Repeated measures correlation coefficients between EORTC domains and EQ-5D-5L in the estimation set (n_patient_ = 609, n_observation_ = 1,269)

|  | **EORTC QLQ-C30** | | | | | | **EORTC CAT CORE** | | | | | |
| --- | --- | --- | --- | --- | --- | --- | --- | --- | --- | --- | --- | --- |
|  | **EQ-5D-5L utility index** | **EQ-5D-5L dimensions** | | | | | **EQ-5D-5L utility index** | **EQ-5D-5L dimensions** | | | | |
|  |  | **Mobility** | **Self-care** | **Usual activities** | **Pain/**  **discomfort** | **Anxiety/**  **depression** |  | **Mobility** | **Self-care** | **Usual activities** | **Pain/**  **discomfort** | **Anxiety/**  **depression** |
| Global health status/QoL | 0.33 | -0.25 | -0.23 | -0.29 | -0.25 | -0.13 | -0.12 | -0.15 | -0.16 | -0.18 | -0.17 | -0.07 |
| Functioning subscales |  |  |  |  |  |  |  |  |  |  |  |  |
| Physical functioning | 0.47 | -0.41 | -0.32 | -0.47 | -0.35 | -0.24 | -0.24 | -0.35 | -0.26 | -0.43 | -0.29 | -0.22 |
| Role functioning | 0.28 | -0.33 | -0.22 | -0.37 | -0.23 | -0.18 | -0.18 | -0.33 | -0.20 | -0.41 | -0.26 | -0.19 |
| Emotional functioning | 0.33 | -0.18 | -0.16 | -0.26 | -0.21 | -0.38 | -0.38 | -0.17 | -0.13 | -0.26 | -0.21 | -0.36 |
| Cognitive functioning | 0.16 | -0.12 | -0.12 | -0.18 | -0.13 | -0.18 | -0.18 | -0.13 | -0.12 | -0.20 | -0.17 | -0.18 |
| Social functioning | -0.31 | -0.24 | -0.23 | -0.36 | -0.18 | -0.23 | -0.23 | -0.21 | -0.18 | -0.31 | -0.17 | -0.25 |
| Symptom scales/items |  |  |  |  |  |  |  |  |  |  |  |  |
| Fatigue | -0.31 | 0.26 | 0.18 | 0.33 | 0.23 | 0.22 | 0.22 | 0.26 | 0.19 | 0.35 | 0.22 | 0.23 |
| Nausea and vomiting | -0.19 | 0.13 | 0.10 | 0.20 | 0.11 | 0.13 | 0.13 | 0.11 | 0.09 | 0.21 | 0.13 | 0.16 |
| Pain | -0.40 | 0.26 | 0.21 | 0.30 | 0.45 | 0.09 | 0.09 | 0.27 | 0.22 | 0.32 | 0.45 | 0.14 |
| Dyspnea | -0.24 | 0.24 | 0.13 | 0.31 | 0.16 | 0.22 | 0.22 | 0.21 | 0.14 | 0.29 | 0.15 | 0.22 |
| Insomnia | -0.18 | 0.12 | 0.10 | 0.16 | 0.17 | 0.17 | 0.17 | 0.14 | 0.10 | 0.15 | 0.18 | 0.17 |
| Appetite loss | -0.21 | 0.14 | 0.13 | 0.19 | 0.12 | 0.10 | 0.10 | 0.14 | 0.10 | 0.19 | 0.12 | 0.10 |
| Constipation | -0.07 | 0.05 | 0.08 | 0.13 | 0.10 | 0.01 | 0.01 | 0.07 | 0.08 | 0.11 | 0.10 | 0.03 |
| Diarrhea | -0.14 | 0.13 | 0.15 | 0.08 | 0.05 | 0.03 | 0.03 | 0.11 | 0.13 | 0.08 | 0.05 | 0.01 |
| Financial difficulties | -0.14 | 0.11 | 0.12 | 0.15 | 0.10 | 0.12 | 0.12 | 0.11 | 0.11 | 0.14 | 0.10 | 0.09 |

The repeated measures correlation coefficients (r_rm_) were estimated by using analysis of covariate (ANCOVA) accounting for inter-individual variability in the R package “*rmcorr*”. The r_rm_ coefficient ranges between -1 to 1 and represents the strength of the linear association between the EORTC domains and the EQ-5D-5L domains.

**
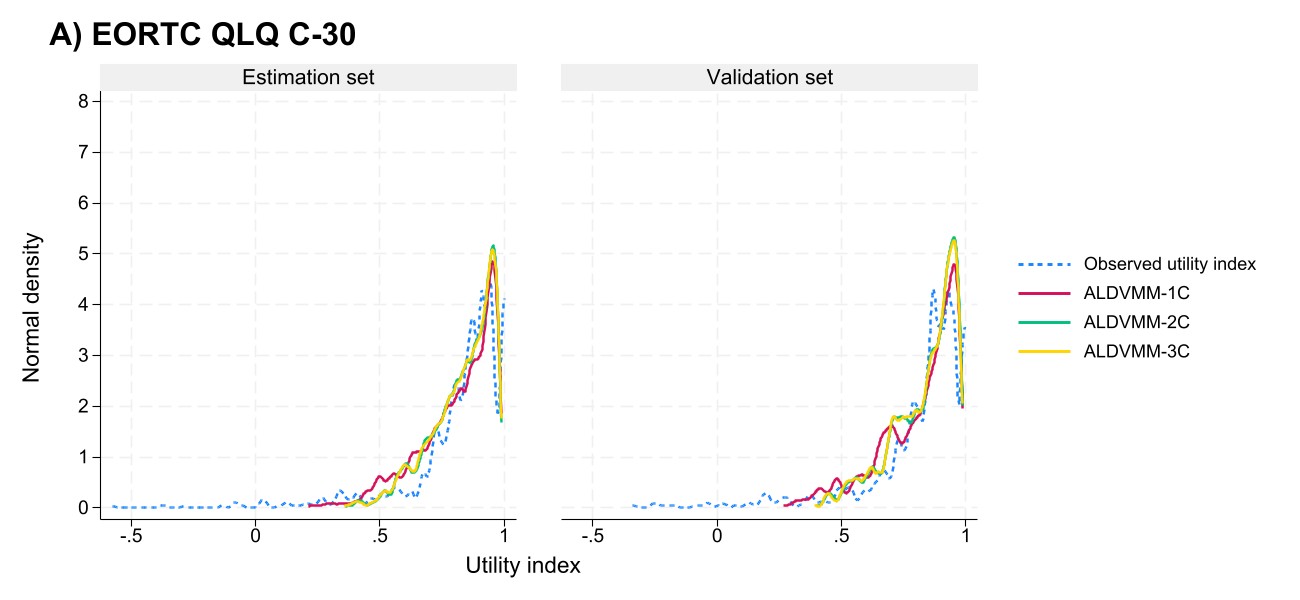
**

**
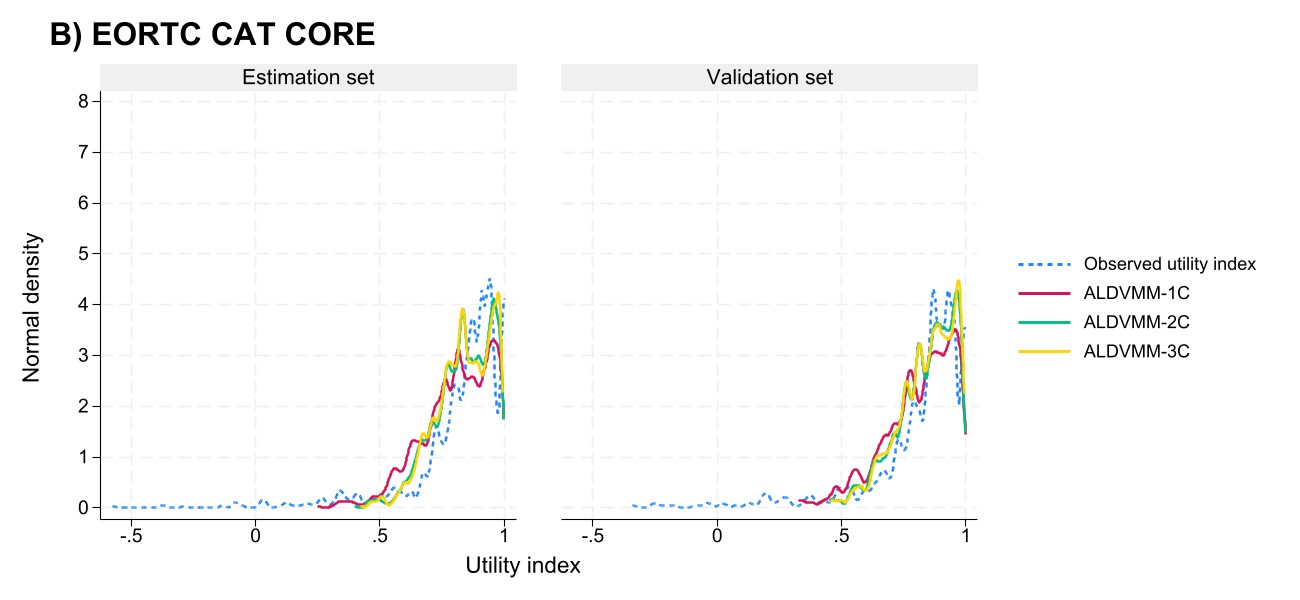
**

**Figure S1** Comparison the density distributions between observed and predicted utility index values between the ALDVMM components. ALDVMM= adjusted limited dependent variable mixture models

**
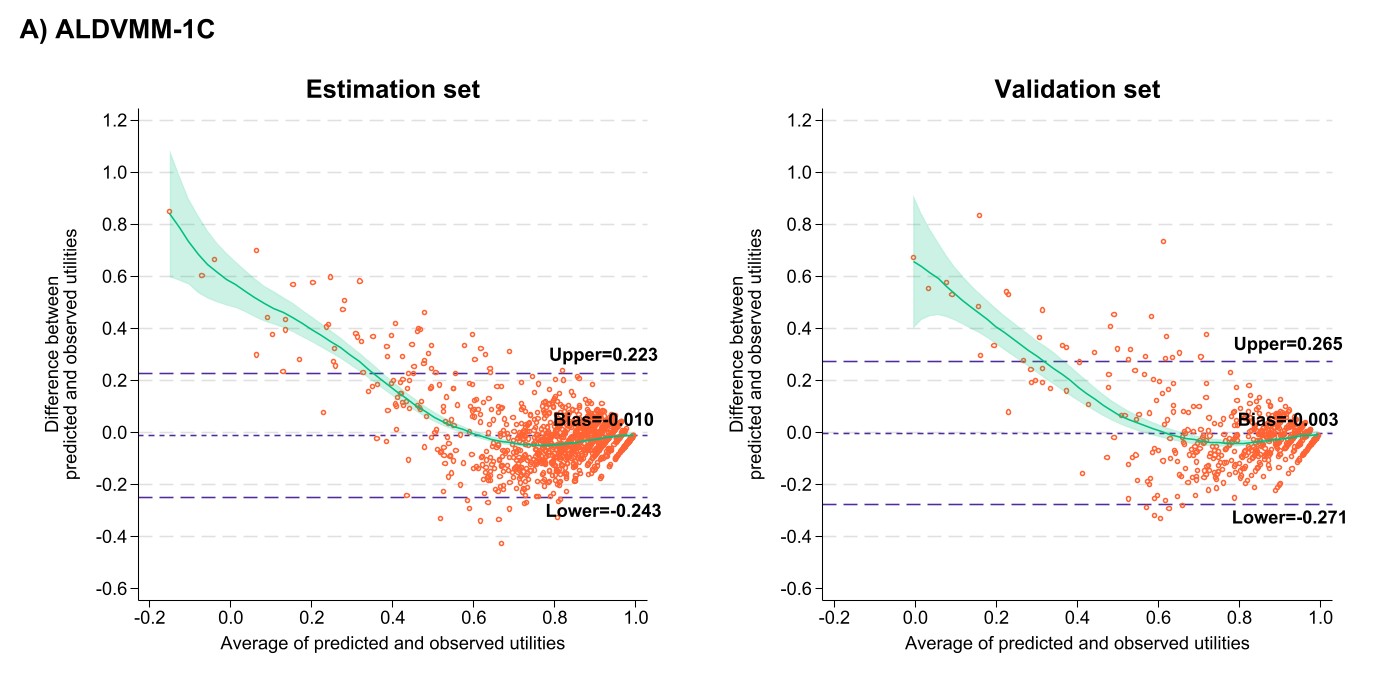
**

**
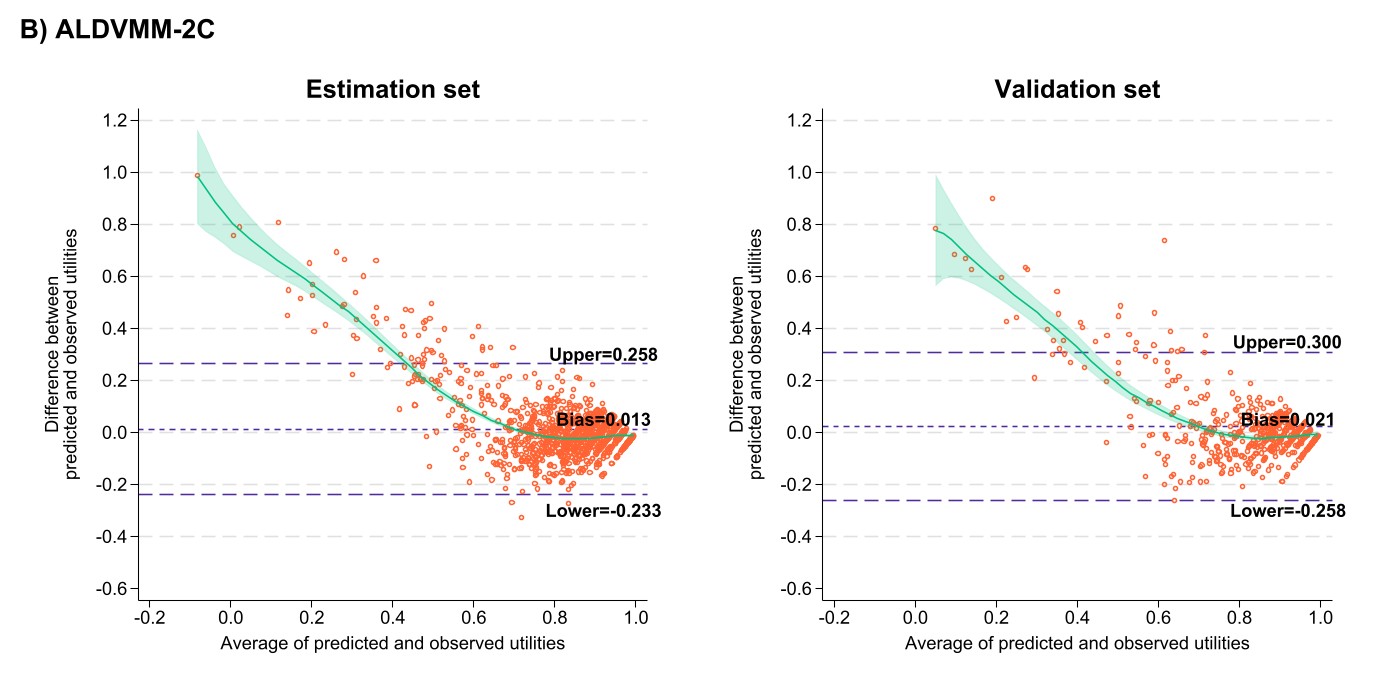
**

**
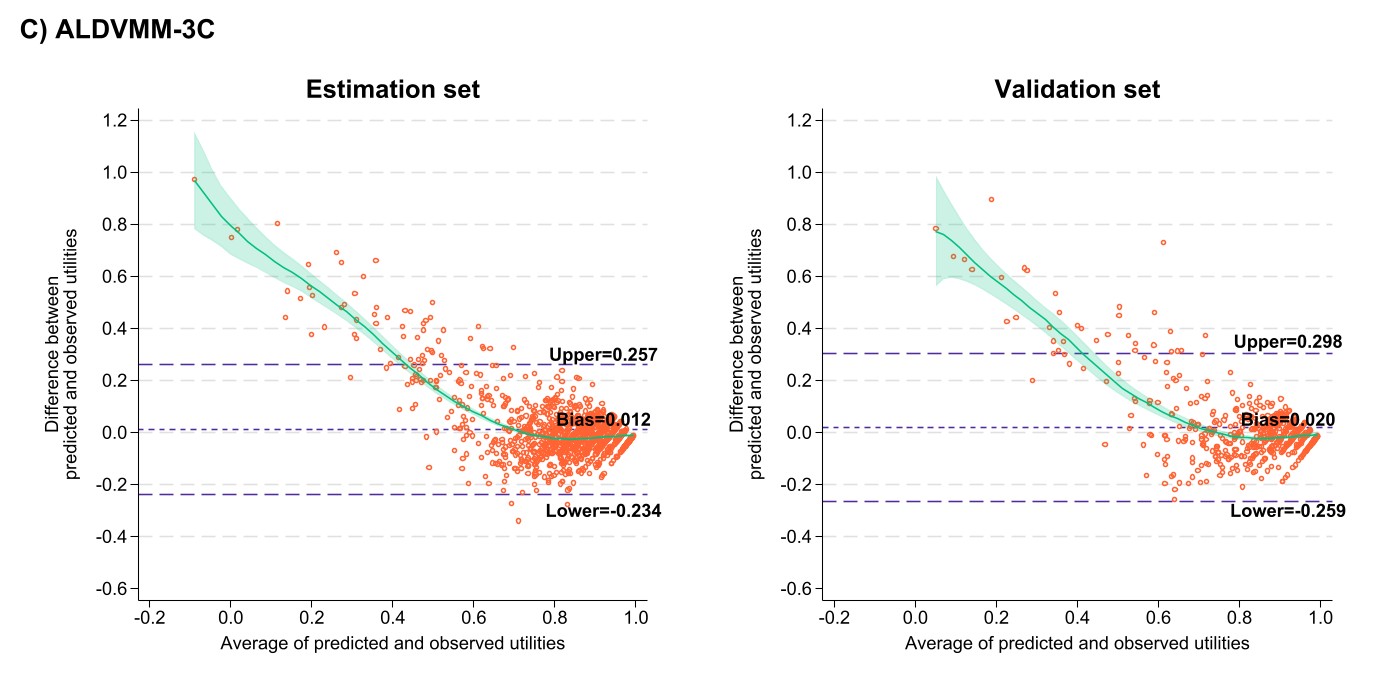
**

**
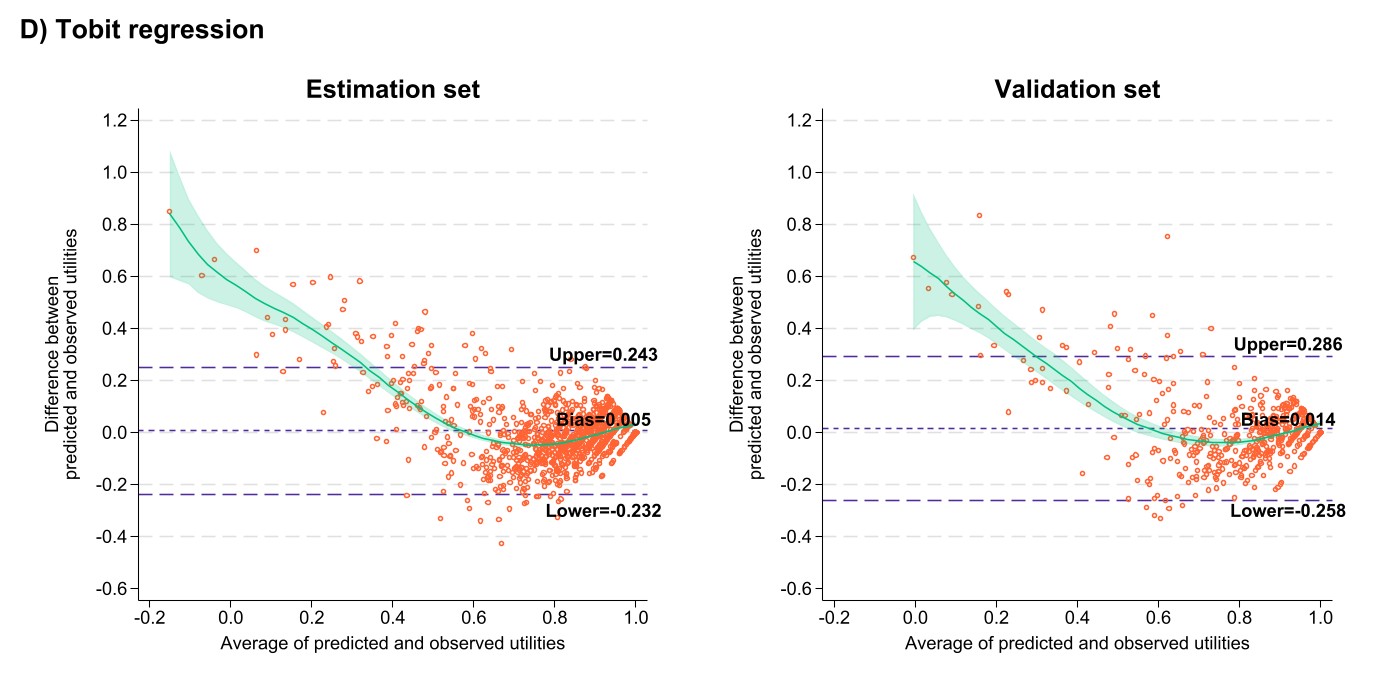
**

**
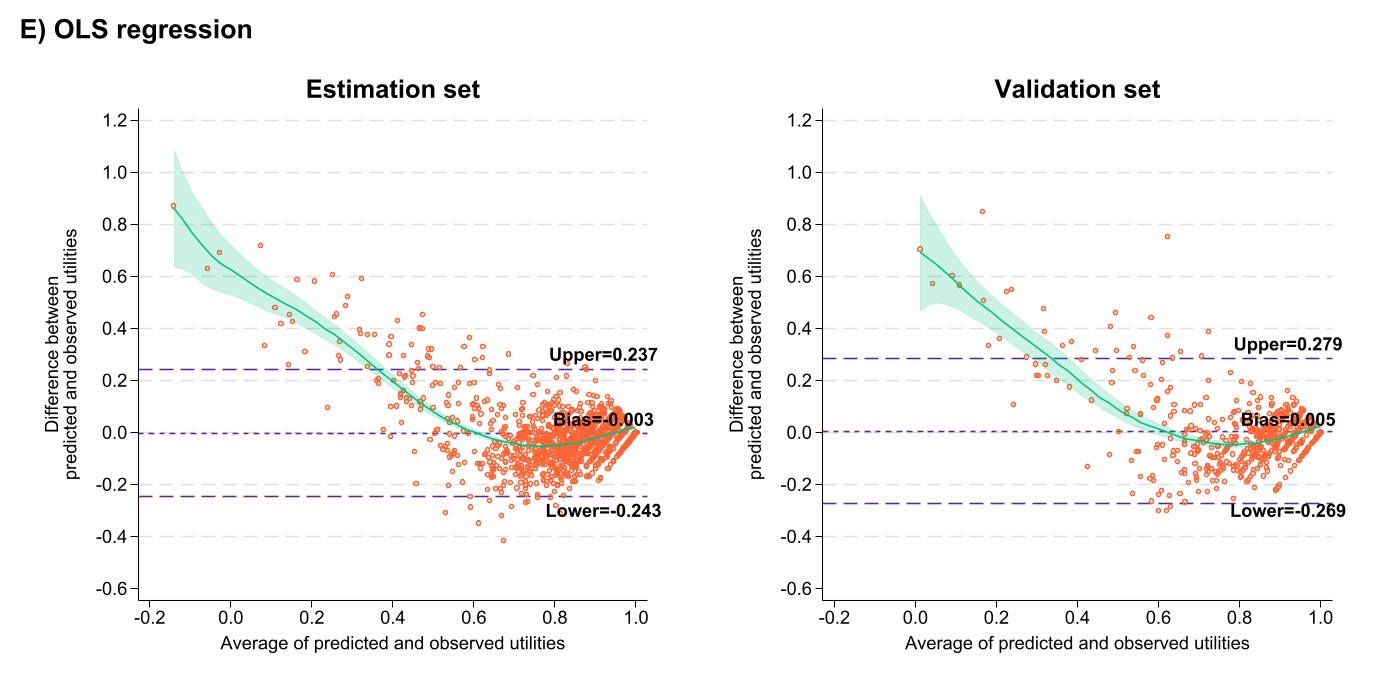
**

**Figure S2** Bland-Altman plot of the observed and predicted mean differences in utility index values from the direct mapping models for the EORTC QLQ-C30. The light green line represents a LOWESS smoothing curve with a 95% confidence interval.

**
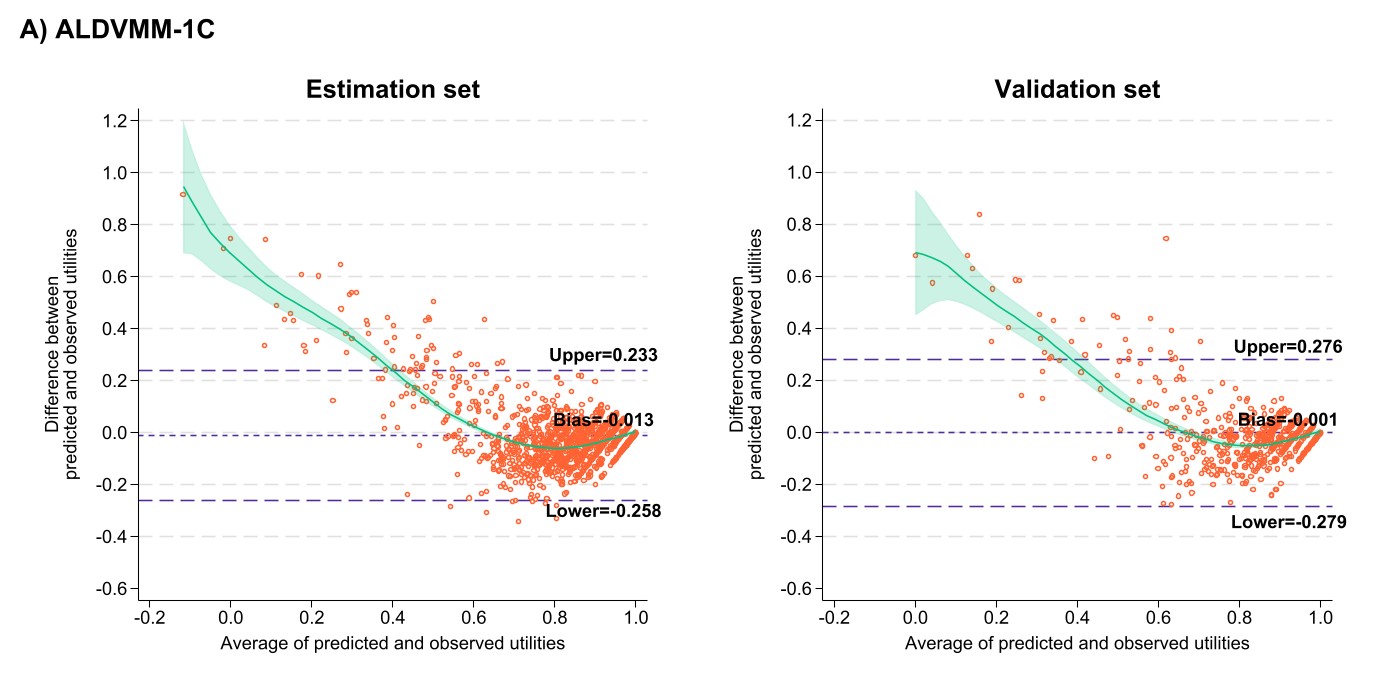
**

**
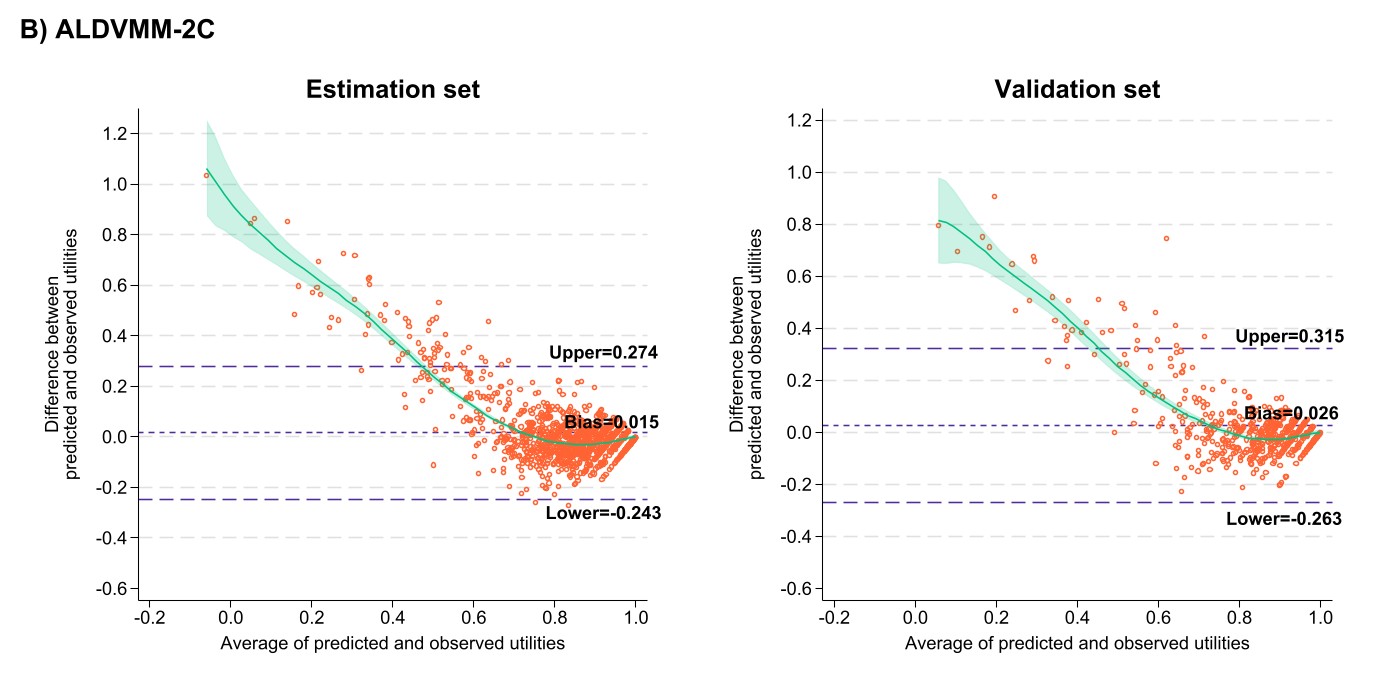
**

**
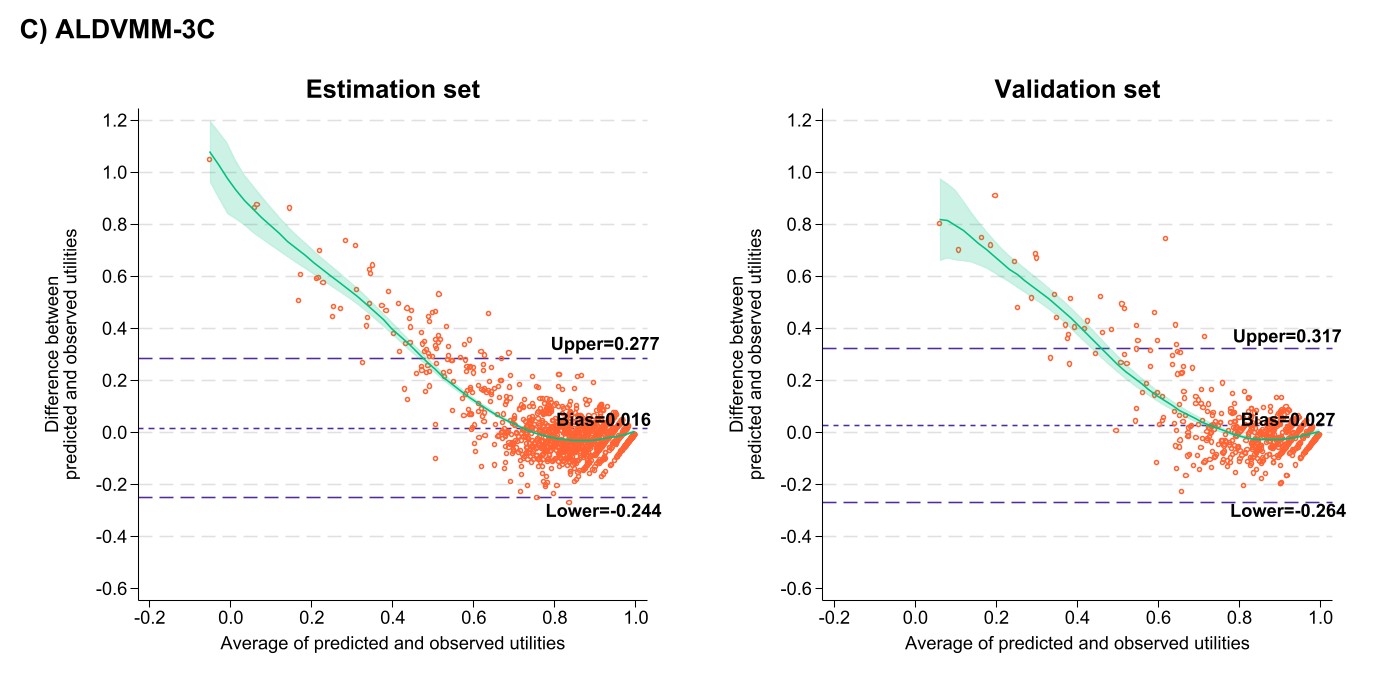
**

**
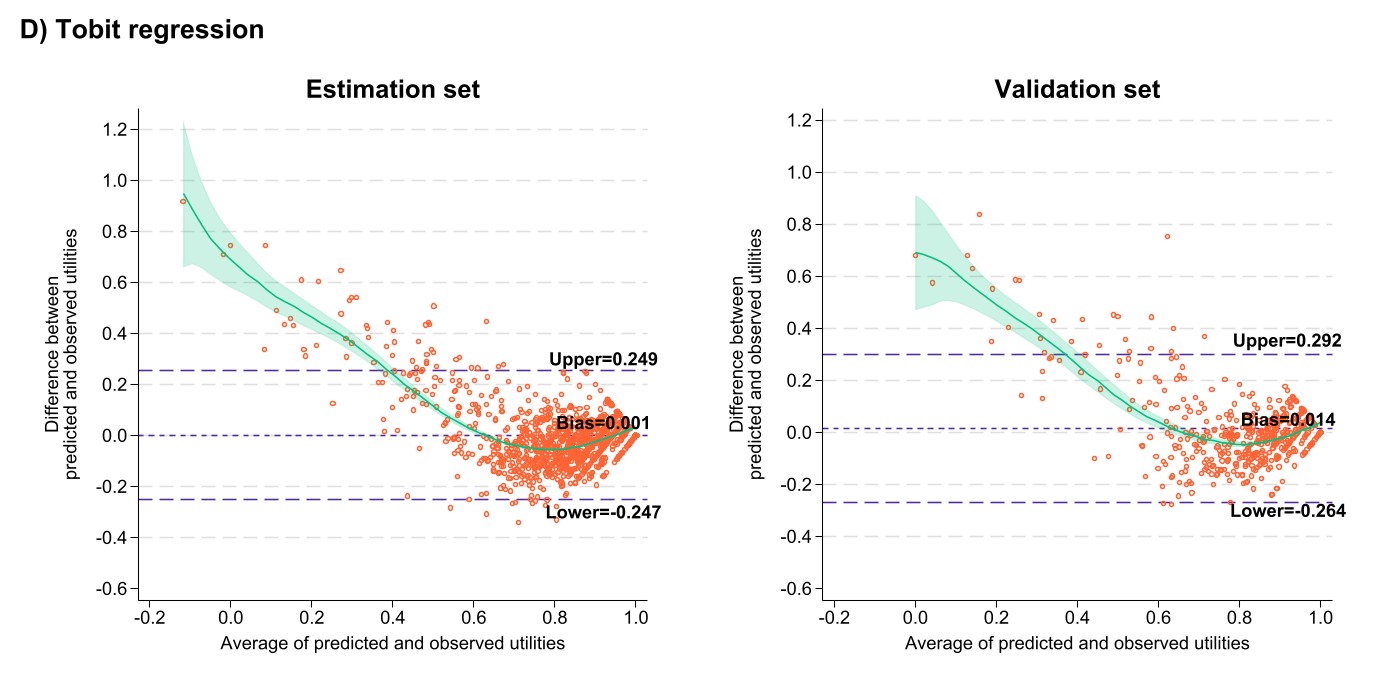
**

**
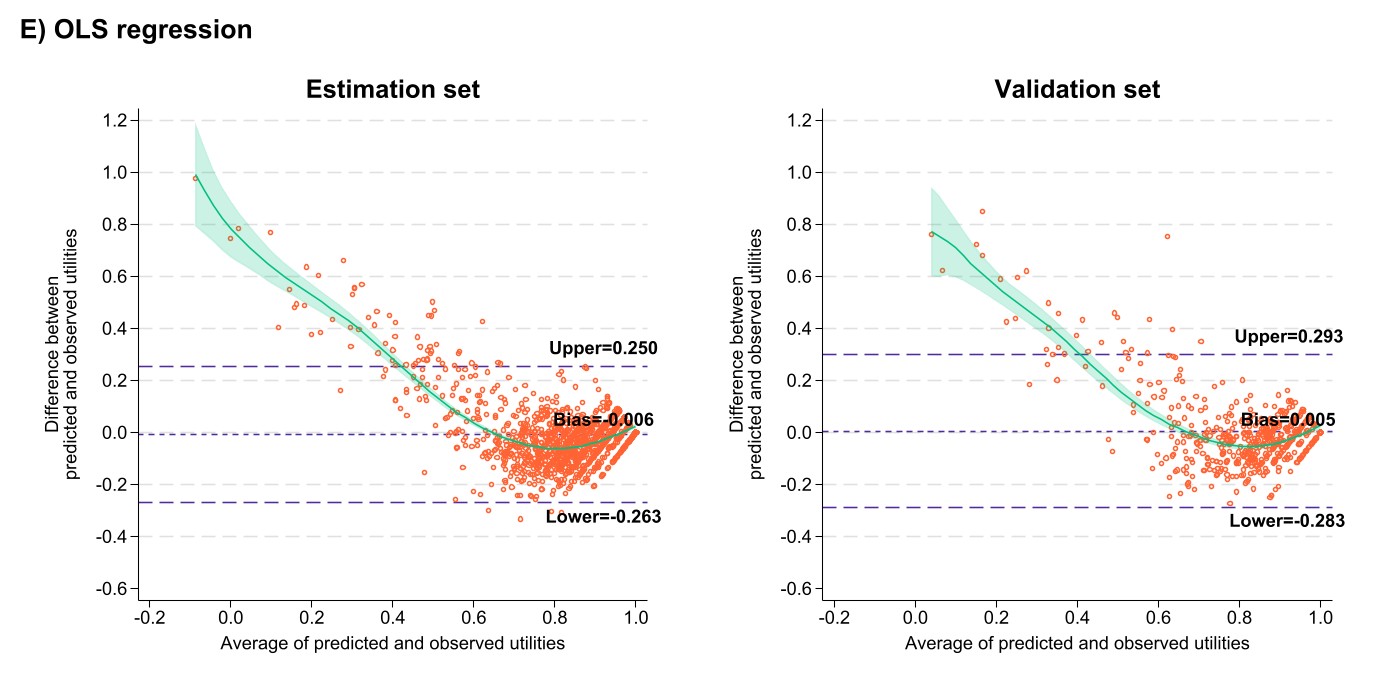
**

**Figure S3** Bland-Altman plot of the observed and predicted mean differences in utility index values from the direct mapping models for the EORTC CAT Core. The light green line represents a LOWESS smoothing curve with a 95% confidence interval.

**Direct and indirect mapping algorithms for EQ-5D-5L**

**Section 1: Direct mapping - Adjusted beta regression**

1. Calculate the utility score

2.1 Linear predictor (η) can be expressed as:

$$\eta= \beta_{0}+\beta_{1}X_{1}+ \beta_{\ldots}X_{\ldots}+ \beta_{n}X_{n}$$

η = predicted linear values

β_0_ = Coefficient of the intercept

β_i_ = Coefficient of the domain (X_i_) from the EORTC, while *i* = 1, …, *n*

2.2 In adjusted beta regression, the expected value 𝐸(𝑦) is typically linked to the linear predictor 𝜂 through a logit link function. This transformation ensures that the predicted values stay within the (0, 1) interval appropriate for beta-distributed outcomes.

The transformed prediction 𝜇 can be obtained as:

​ $\mu= \frac{1}{1+ e^{-\eta}}$

1. Back-transform predicted values

$$Y_{predict}= \mu*(1+0.661))-0.661$$

Predicted utility values need to be back-transformed since the EQ-5D-5L utilities in the German tariff has a lowest score -0.661, we transformed the utilities to the interval (0, 1) by $Y_{trans}= \frac{(Y+0.661)}{(1+0.661)}$ before fitting the model.

**Table S4** Performance of the adjusted beta regression models for predicting the utilities from the estimation set for metastasis breast cancer (n_patient_ = 609, n_observation_ = 1,269)

|  | **EORTC QLQ C-30** | | **EORTC CAT Core** | |
| --- | --- | --- | --- | --- |
|  | **β** | **SE** | **β** | **SE** |
| Intercept | 0.00022 | 0.20698 | -122.147 | 0.64104 |
| Global heath/QoL | 0.00816 | 0.00163 | 0.03934 | 0.00937 |
| Physical functioning | 0.01822 | 0.00158 | 0.04033 | 0.00424 |
| Role functioning | 0.00261 | 0.00157 | 0.01383 | 0.00426 |
| Emotional functioning | 0.00678 | 0.00130 | 0.01795 | 0.00390 |
| Cognitive functioning | 0.00235 | 0.00112 | 0.00701 | 0.00333 |
| Social functioning | 0.00018 | 0.00146 | 0.00464 | 0.00463 |
| Fatigue | 0.00335 | 0.00124 | 0.00985 | 0.00401 |
| Nausea and vomitting | -0.00077 | 0.00148 | -0.00456 | 0.00251 |
| Pain | -0.00937 | 0.00084 | -0.03214 | 0.00310 |
| Dyspnea | 0.00006 | 0.00087 | 0.00349 | 0.00268 |
| Insomnia | -0.00004 | 0.00079 | 0.00266 | 0.00307 |
| Appetite loss | 0.00042 | 0.00083 | 0.00189 | 0.00224 |
| Constipation | 0.00006 | 0.00086 | 0.00007 | 0.00226 |
| Diarrhea | -0.00001 | 0.00080 | 0.00017 | 0.00200 |
| Financial difficulties | -0.00231 | 0.00075 | -0.00737 | 0.00210 |
| AIC | -4450.65 |  | -4472.73 |  |
| BIC | -4363.17 |  | -4385.25 |  |

AIC, Akaike information criterion; BIC, Bayesian information criterion; β, unstandardized coefficient; SE, standard error adjusted for 609 clusters (patients); QoL, quality of life

**Stata code for direct mapping**

***predict utilities from EORTC QLQ C-30**

**//Estimate linear predictor** (η)

0.00022 + 0.00816*scoresql ///

+ 0.01822*scorespf ///

+ 0.00261*scoresrf ///

+ 0.00678*scoresef ///

+ 0.00235*scorescf ///

+ 0.00018*scoressf ///

+ 0.00335*scoresfa ///

- 0.00077*scoresnv ///

- 0.00937*scorespa ///

+ 0.00006*scoresdy ///

- 0.00004*scoressl ///

+ 0.00042*scoresap ///

+ 0.00006*scoresco ///

- 0.00001*scoresdi ///

- 0.00231*scoresfi

**// transformed prediction 𝜇**

gen calc_beta_predic_trans1 = 1/(1 + exp(-calc_beta_predic_trans))

**// Back-transform predicted values**

gen calc_beta_predic = (calc_beta_predic_trans1 * (1 + 0.594)) - 0.594

***predict utilities from EORTC CAT Core *T*-score**

gen calc_beta_predic_ib_trans = -1.22147 + 0.03934*scoresql_itembank ///

+ 0.04033*scorespf_itembank ///

+ 0.01383*scoresrf_itembank ///

+ 0.01795*scoresef_itembank ///

+ 0.00701*scorescf_itembank ///

+ 0.00464*scoressf_itembank ///

+ 0.0085*scoresfa_itembank ///

- 0.00456*scoresnv_itembank ///

- 0.03214*scorespa_itembank ///

+ 0.00349*scoresdy_itembank ///

+ 0.00266*scoressl_itembank ///

+ 0.00189*scoresap_itembank ///

+ 0.00007*scoresco_itembank ///

+ 0.00017*scoresdi_itembank ///

- 0.00737*scoresfi_itembank

**// transformed prediction 𝜇**

gen calc_beta_predic_ib_trans1 = 1/(1 + exp(-calc_beta_predic_ib_trans))

**// Back-transform predicted values**

gen calc_beta_predic_ib = (calc_beta_predic_ib_trans1 * (1 + 0.594)) - 0.594

**Section 2: Indirect mapping - Generalized ordered logit model**

**Estimating EQ-5D-5L for each dimension**

The EQ-5D-5L system measures health status across five dimensions: Mobility, Self-care, Usual Activities, Pain/Discomfort, and Anxiety/Depression. Each dimension can have one of five levels of problems: No Problems, Slight Problems, Moderate Problems, Severe Problems, and Extreme Problems.

Table S5 and table S6 provide **coefficients** and **intercepts** for each dimension and each level. Please see the full Stata code in “*Supplementary_Stata code_Indirect mapping_C30.do*” and “*Supplementary_Stata code_Indirect mapping_CAT Core.do*”.

To predict the probability of being in a particular level for a given dimension (e.g., Mobility), you will follow these steps:

1. **Calculate the linear part**

For example,

$$est_{linear}=-0.0173\boldsymbol{QL}-0.0715\boldsymbol{PF}-0.0082\boldsymbol{RF}+0.0040\boldsymbol{EF}+0.0016\boldsymbol{CF}+0.0012\boldsymbol{SF}-0.0109\boldsymbol{FA}+0.0007\boldsymbol{NV}+0.0131\boldsymbol{PA}+0.0041\boldsymbol{DY}-0.0020\boldsymbol{SL}-0.0022\boldsymbol{AP}-0.0005\boldsymbol{CO}-0.0016\boldsymbol{DI}+0.0010\boldsymbol{FI}$$

1. **Calculate the logit (log-odds)** for each outcome level (No Problems, Slight Problems, Moderate Problems, Severe Problems). Each level has a unique intercept and uses the same coefficients for the predictors.

For example,

$$logit\left( P\left( Mobility\leq\text{No problems} \right) \right)=6.2805+ {est}_{linear}$$

$$logit\left( P\left( Mobility\leq\text{Slight problems} \right) \right)=4.3874+ {est}_{linear}$$

$$logit\left( P\left( Mobility\leq\text{Moderate problems} \right) \right)=2.1653+ {est}_{linear}$$

$$logit\left( P\left( Mobility\leq\text{Severe problems} \right) \right)=-1.2445+ {est}_{linear}$$

1. **Convert the logits to probabilities** using the logistic function.

$$P\left( Y\leq j \right)=\frac{1}{1+e^{-(logit(P\left( Y\leq j \right))}}$$

When *j* = no, slight, moderate, severe problems and Y = dimensions, e.g., mobility

1. **Calculate the probability of being in each specific level** by subtracting the cumulative probabilities for adjacent levels.

P(No problems) = 1 – P(No problems)

P(Slight problems) = P (No problems) – P(Slight problems)

P(Moderate problems) = P(Slight problems) – P(Moderate problems)

P(Severe problems) = P(Moderate problems) – P(Severe problems)

P(Extreme problems) = P(Severe problems)

**Table S5** Estimated coefficients and standard error of the indirect mapping model for the EORTC QLQ C-30 from the estimation set (n_patient_ = 609, n_observation_ = 1,269)

| **EORTC QLQ C-30 Scores** | **EQ-5D-5L dimensions – β (SE)** | | | | |
| --- | --- | --- | --- | --- | --- |
|  | **Mobility** | **Self-care** | **Usual activities** | **Pain/discomfort** | **Anxiety/depression** |
| Global heath/QoL (QL) | -0.0173 (0.0046) | -0.0274 (0.0067) | -0.0237 (0.0044) | -0.0236 (0.0044) | -0.0159 (0.0042) |
| Physical functioning (PF) | -0.0715 (0.0048) | -0.0730 (0.0066) | -0.0555 (0.0046) | -0.0296 (0.0045) | -0.0050 (0.0043) |
| Role functioning (RF) | -0.0082 (0.0039) | -0.0070 (0.0051) | -0.0205 (0.0038) | -0.0096 (0.0039) | 0.0076 (0.0038) |
| Emotional functioning (EF) | 0.0040 (0.0037) | 0.0062 (0.0054) | 0.0045 (0.0036) | -0.0007 (0.0035) | -0.0560 (0.0038) |
| Cognitive functioning (CF) | 0.0016 (0.0032) | -0.0136 (0.0044) | -0.0084 (0.0031) | -0.0036 (0.0032) | -0.0107 (0.0030) |
| Social functioning (SF) | 0.0012 (0.0034) | -0.0054 (0.0046) | -0.0100 (0.0032) | 0.0106 (0.0033) | -0.0073 (0.0032) |
| Fatigue (FA) | -0.0109 (0.0039) | -0.0147 (0.0056) | 0.0086 (0.0038) | -0.0071 (0.0038) | -0.0084 (0.0037) |
| Nausea and vomitting (NV) | 0.0007 (0.0036) | -0.0020 (0.0044) | 0.0013 (0.0035) | 0.0028 (0.0038) | 0.0073 (0.0034) |
| Pain (PA) | 0.0131 (0.0026) | -0.0010 (0.0034) | 0.0032 (0.0025) | 0.0582 (0.0032) | -0.0025 (0.0025) |
| Dyspnea (DY) | 0.0041 (0.0025) | -0.0061 (0.0034) | 0.0006 (0.0024) | -0.0020 (0.0025) | -0.0041 (0.0024) |
| Insomnia (SL) | -0.0020 (0.0023) | -0.0034 (0.0032) | -0.0037 (0.0022) | 0.0004 (0.0022) | 0.0022 (0.0021) |
| Appetite loss (AP) | -0.0022 (0.0026) | 0.0100 (0.0034) | 0.0001 (0.0025) | 0.0008 (0.0026) | -0.0027 (0.0025) |
| Constipation (CO) | -0.0005 (0.0022) | 0.0060 (0.0030) | -0.0028 (0.0022) | 0.0002 (0.0023) | -0.0015 (0.0022) |
| Diarrhea (DI) | -0.0016 (0.0023) | 0.0015 (0.0030) | -0.0046 (0.0022) | -0.0028 (0.0022) | -0.0042 (0.0021) |
| Financial difficulties (FI) | 0.0010 (0.0023) | 0.0052 (0.0030) | 0.0022 (0.0022) | 0.0030 (0.0023) | 0.0040 (0.0022) |
| **Intercepts for each level** |  |  |  |  |  |
| No problems | 6.2805 (0.6367) | 6.3057 (0.8647) | 8.6367 (0.6468) | 4.6352 (0.6202) | 6.4233 (0.6126) |
| Slight Problems | 4.3874 (0.6220) | 4.2266 (0.8409) | 5.8714 (0.6187) | 0.9696 (0.6076) | 3.9806 (0.5914) |
| Moderate problems | 2.1653 (0.6082) | 2.2664 (0.8381) | 3.0518 (0.5964) | -2.5259 (0.6142) | 1.8011 (0.5857) |
| Severe problems | -1.2445 (0.7093) | 0.3540 (0.9374) | 0.2041 (0.6210) | -5.7409 (0.6977) | -0.8218 (0.6817) |
| **AIC** | 2145.14 | 1009.40 | 2177.12 | 2166.59 | 2382.72 |
| **BIC** | 2243.21 | 1107.47 | 2275.19 | 2264.66 | 2480.79 |

AIC, Akaike information criterion; BIC, Bayesian information criterion; β, unstandardized coefficient; SE, standard error ; QoL, quality of life

**Table S6** Estimated coefficients and standard error of the indirect mapping model for the EORTC CAT Core *T*-score from the estimation set (n_patient_ = 609, n_observation_ = 1,269)

| **EORTC CAT CORE *T*-score** | **EQ-5D-5L dimensions – β (SE)** | | | | |
| --- | --- | --- | --- | --- | --- |
|  | **Mobility** | **Self-care** | **Usual activities** | **Pain/discomfort** | **Anxiety/depression** |
| Global heath/QoL (QL) | -0.0600 (0.0354) | -0.0710 (0.0421) | -0.0947 (0.0373) | -0.0935 (0.0250) | -0.0738 (0.0229) |
| Physical functioning (PF) | -0.1889 (0.0164) | -0.1879 (0.0213) | -0.1114 (0.0136) | -0.0550 (0.0133) | -0.0193 (0.0140) |
| Role functioning (RF) | -0.0312 (0.0130) | -0.0537 (0.0178) | -0.1170 (0.0151) | -0.0303 (0.0127) | 0.0206 (0.0122) |
| Emotional functioning (EF) | 0.0151 (0.0118) | 0.0117 (0.0176) | 0.0045 (0.0113) | 0.0053 (0.0106) | -0.1708 (0.0129) |
| Cognitive functioning (CF) | -0.0148 (0.0097) | -0.0204 (0.0147) | -0.0362 (0.0091) | -0.0093 (0.0088) | -0.0205 (0.0093) |
| Social functioning (SF) | -0.0173 (0.0118) | -0.0452 (0.0180) | -0.0216 (0.0113) | 0.0122 (0.0108) | -0.0375 (0.0129) |
| Fatigue (FA) | -0.0246 (0.0125) | -0.0496 (0.0173) | 0.0185 (0.0125) | -0.0230 (0.0127) | -0.0421 (0.0129) |
| Nausea and vomitting (NV) | -0.0015 (0.0074) | 0.0013 (0.0091) | 0.0092 (0.0075) | 0.0116 (0.0082) | 0.0209 (0.0068) |
| Pain (PA) | 0.0426 (0.0107) | 0.0198 (0.0126) | 0.0107 (0.0088) | 0.1973 (0.0133) | -0.0040 (0.0090) |
| Dyspnea (DY) | -0.0104 (0.0089) | -0.0087 (0.0124) | -0.0022 (0.0085) | -0.0155 (0.0083) | -0.0066 (0.0091) |
| Insomnia (SL) | -0.0003 (0.0099) | -0.0288 (0.0150) | -0.0271 (0.0092) | 0.0031 (0.0094) | 0.0041 (0.0096) |
| Appetite loss (AP) | -0.0037 (0.0071) | -0.0010 (0.0102) | -0.0052 (0.0073) | -0.0041 (0.0074) | -0.0074 (0.0064) |
| Constipation (CO) | 0.0049 (0.0067) | 0.0077 (0.0105) | -0.0055 (0.0067) | 0.0031 (0.0065) | -0.0122 (0.0069) |
| Diarrhea (DI) | -0.0035 (0.0066) | 0.0121 (0.0087) | -0.0091 (0.0061) | -0.0069 (0.0058) | -0.0116 (0.0060) |
| Financial difficulties (FI) | 0.0079 (0.0065) | 0.0303 (0.0094) | 0.0160 (0.0068) | 0.0050 (0.0073) | 0.0167 (0.0063) |
| **Intercepts for each level** |  |  |  |  |  |
| No problems | 11.2775 (2.3687) | 13.1350 (2.8343) | 15.6764 (2.1814) | -0.0481 (2.1487) | 15.3226 (2.0702) |
| Slight Problems | 9.4303 (2.3620) | 11.1133 (2.8050) | 12.8686 (2.1630) | -3.9541 (2.1755) | 12.8804 (2.0662) |
| Moderate problems | 7.2531 (2.3723) | 9.4032 (2.7679) | 10.0714 (2.1507) | -7.1464 (2.1901) | 10.8377 (2.0668) |
| Severe problems | 2.0368 (2.6591) | 7.4605 (2.7323) | 7.2427 (2.1568) | -10.0359 (2.2225) | 8.6868 (2.0932) |
| **AIC** | 2073.41 | 1015.73 | 2062.71 | 2091.56 | 2331.68 |
| **BIC** | 2171.19 | 1113.50 | 2160.49 | 2189.34 | 2429.45 |

AIC, Akaike information criterion; BIC, Bayesian information criterion; β, unstandardized coefficient; SE, standard error ; QoL, quality of life
